# Nitrogen-Doped Carbon Encapsulation of Fe Composites: Synthesis Tuning Their Physical Properties for Syngas Conversion to Valuable Compounds

**DOI:** 10.1021/acsomega.6c05461

**Published:** 2026-08-19

**Authors:** João Pedro S. Nascimento, Alcineia C. Oliveira, Fabiano A. N. Fernandes, Gardênia S. Pinheiro, Gilberto D. Saraiva, Ramon R. Peña-Garcia, Marco A. Morales Torres, José Jiménez-Jiménez, Enrique Rodríguez-Castellón

**Affiliations:** † 28121 Universidade Federal do Ceará, Departamento de Química Analítica e Físico-Química, Campus do Pici-Bloco 940, Fortaleza 60355-636, Ceará, Brazil; ‡ Universidade Federal do Ceará, Departamento de Engenharia Química, Campus do Pici-Bloco 709, Fortaleza 60355-636, Ceará, Brazil; § Universidade Federal do Piauí, Departamento de Fisica, Campus Universitário Ministro Petrônio Portella - Bairro Ininga, Teresina, Piauí 64049-550, Brazil; ∥ Universidade Estadual do Ceará, Faculdade de Educação, Ciências e Letras do Sertão Central, Rua José de Queiroz Pessoa, Planalto Universitário, Quixadá 60740-000, Ceará, Brazil; ⊥ 67744 Universidade Federal de Pernambuco, Rua Cento e Sessenta e Três, Unidade Acadêmica do Cabo de Santo Agostinho, Recife 3512-580, Pernambuco, Brazil; # 28123 Universidade Federal do Rio Grande do Norte, Departamento de Física Teórica e Experimental, Av. Sen. Salgado Filho, Natal 59078-970, Rio Grande do Norte, Brazil; ∇ 16752 Universidad de Málaga, Facultad de Ciencias, Departamento de Química Inorgánica, Cristalografía y Mineralogía, Instituto Interuniversitario de Investigación en Biorrefinerías I3B, Málaga 29071, Spain

## Abstract

Nitrogen-doped carbon encapsulation of Fe composites was achieved through polymer-assisted coprecipitation synthesis with urea. SEM-EDS, XRD, HRTEM, Raman, and FTIR spectroscopy revealed nitrogen-doped graphitic carbon layers encapsulating the Fe spinel phase (γ-Fe_2_O_3_, Fe_3_O_4_) or other spinel-related phases. The key innovation lies in the design of nitrogen-doped carbon-supported Fe composites with the addition of heteroatoms, e.g., Fe_*x*_NC/MFe (M = Li, Mg, Al, Ti, Co, or Ni), considerably affecting their catalytic performances in syngas conversion via Fischer–Tropsch reaction. A novel aspect of these solids was revealed by XPS and textural properties results that showed the modulation of the porous structure of the composites by the existence of surface iron carbides and Fe_3_N iron nitrides covering a carbon matrix strongly bonded to the Fe^2+^/Fe^3+^ entities from the Fe_*x*_NC/NiFe. Mössbauer and Raman spectroscopy and XRD confirmed the presence of a bulk structure consisting of spinel ferrites, besides the introduction of defects into the structure. These facts enhanced the syngas conversion to 44% and improved the selectivity toward light and heavy hydrocarbons in the FTS reaction at a pressure of 20 atm, 270 °C, and CO/H_2_ molar ratio of 1.

## Graphical abstract

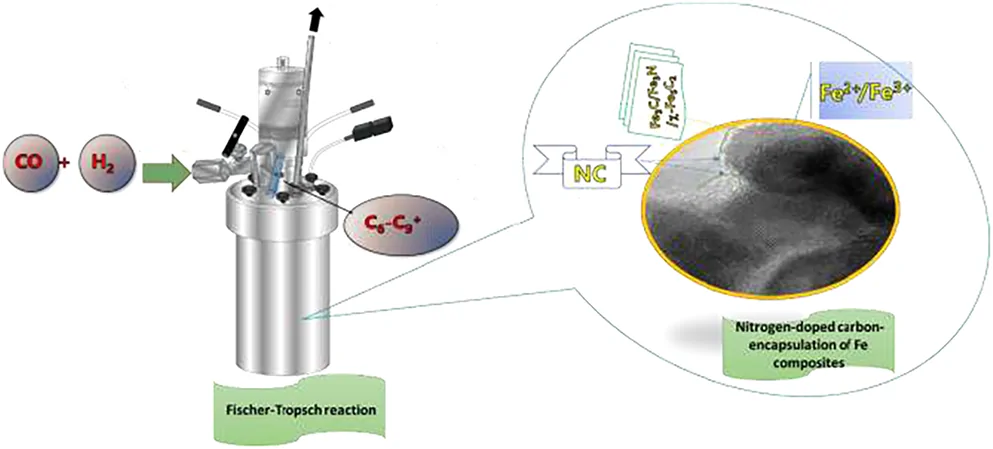

## 1 Introduction

The advances in the synthesis of nitrogen-doped carbon-based materials are particularly relevant owing to their several key advantages in the field of electrochemical, catalytic, and adsorption performances.^1–3^ Of importance, the nitrogen-doped carbon materials have lone pair electrons, making the N atom more electronegative than the carbon atom.^1,3,4^ This enables a strong interaction with positive particles and high electron density of nitrogen, in contrast to the adjacent carbon of low electronegativity that provides weaker interactions with negative particles.^3,4^ Such features are of fundamental importance for catalysis, as even challenges to modulate their properties by synthetic routes have continued to be persistent.^2^

Especially interesting are the physicochemical properties of nitrogen-doped metal carbonaceous materials. Among them, heteroatom-doped carbon materials, in which metal M = Fe, Co, or Ni, are drawing significant attention because of their favorable redox and acid–base properties.^5–7^ In the case of nitrogen-doped carbon materials encapsulated with Fe composites, the chemical compositions of the species result in structural distortions, changes in the charge density in the carbonaceous structure, and in some cases, synergistic effects, which profoundly modulate the reactivity in electrocatalytic reactions.^5–8^ On the other hand, Fe encapsulation in nitrogen-doped carbon materials having active sites such as C–N_*x*_, Fe_3_C, and Fe–N_*x*_ shows unsatisfactory performance after several cycles attributable to the degradation of the carbon matrix when used as catalysts in dehydrogenation, oxidation, hydrogenation, and coupling processes.^1,6,9^ The encapsulation of FeM nanoparticles in nitrogen-codoped carbon materials confers a distinct benefit of improving the catalytic performance, since the carbonaceous matrix covers the FeM particles from erosion and phase transitions, in addition to the Fe coordinated to nitrogen, or in a FeM alloy, offers promising alternatives in severe catalytic reaction environments.^9,10^

Within this context, synthesis gas, a mixture of CO and H_2_, is one of the most important feedstocks in chemical processes due to its high consumption in many applications, including fuel cells, methanol production, and Fischer–Tropsch synthesis (FTS).^11,12^ The most widely used process for synthesis gas production with a low H_2_/CO ratio is the FTS reaction, aiming to produce olefins, paraffins, and alcohols,^13,14^ according to Figure 1. Also, FTS produces highly desirable lower olefins (C_2_∼C_4_) and high value-added chemicals such as gasoline (C_5_∼C_11_), jet fuels (C_9_∼C_16_), and diesel (C_12_∼C_20_).^15,16^

**Figure 1:**
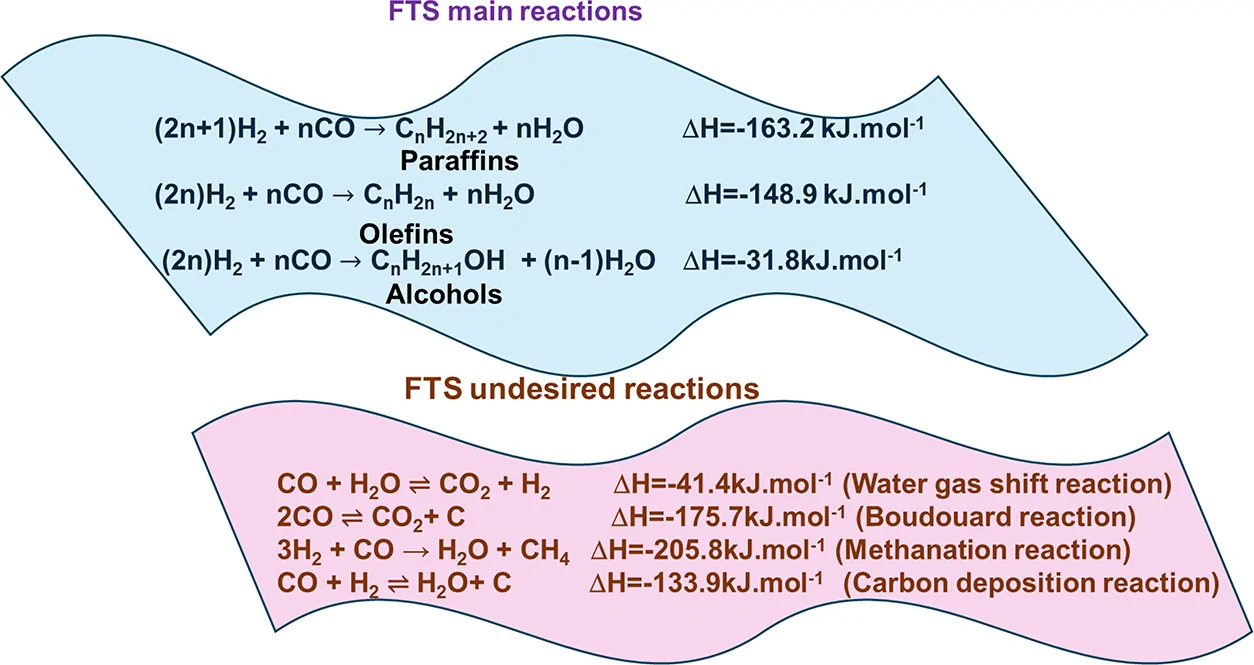
Schematic representation of the syngas conversion by FTS main reactions along with the undesired reactions.

In view of the current climate change and energy crisis, the FTS reaction is considered to be a promising process to tackle the issues for sustainable economic development of clean fuels with low sulfur and aromatic contents from petroleum gas sources.^11,15^

Numerous catalytic systems composed of transition metal catalysts have been evaluated in the FTS reaction, such as iron, cobalt, nickel, and ruthenium, all of them showing high activity.^17–19^ In this scenario, iron and cobalt are commercially available for FTS owing to their low cost, excellent activity, and stability, when compared to their ruthenium counterparts.^20,21^ Bulk or supported iron-based catalysts with various formulations constitute one of the best catalysts for the FTS reaction, regarding the reduced activity for carbon chain elongation, limited hydrogenation reactivity, high selectivity toward light olefins, and enhanced performance in the water–gas shift (WGS) reaction.^20^

However, the oxidation of metallic iron or iron carbide to Fe_3_O_4_ is still the main drawback, limiting their large-scale commercialization in the FTS process.^20–22^ Moreover, the iron-based catalyst deactivation by sintering and coking, along with the high WGS reaction activity and other undesirable FTS reactions (Figure 1) as well as a broad hydrocarbon distribution, restrict the application of the Fe catalysts in the FTS reaction.^11,12^

To overcome these problems, tailoring of the catalyst properties by modulating the nature of the components concomitantly used with iron-based oxide catalysts, changes in the type of support, and the process variables at which the FTS reaction operates, make the development of novel iron-based catalysts an imperative topic.^9,20^ Several attempts to modify the iron-based catalysts using various supports such as Al_2_O_3_, SiO_2_, zeolites, ZrO_2_, TiO_2_, and carbons have been made to improve iron dispersion and thus enhance their activity and stability.^20,22^

In this sense, carbon-based supports offer the advantage of relatively weak interaction with the iron species, in addition to providing a higher Fe species dispersion.^23,24^ Besides, carbon functionality may provide a suitable impact on the FTS activity through hydrocarbon selectivity and possible low methane production.^20^ At present, carbon supports such as carbon spheres, carbon black, activated carbon, mesoporous carbons, graphene, nanofibers, carbon aerogel, metal–organic framework (MOF)-derived carbon, nanotubes, and glassy carbon, among others, are evaluated successfully in FTS reaction with better performance than their oxide support counterparts.^23–26^

Our earlier work has shown that Fe supported on mesoporous carbon possesses a notable surface chemistry, in which the functional surface groups give the Fe species encapsulation inside their porous carbonaceous matrix, which is a factor that determines the efficiency of the solid during the FTS reaction.^15^ Despite the improvement in FTS reaction activity, an opposite trend is observed for the selectivities toward higher-molecular-weight hydrocarbons and production of lower olefins.

Inspired by these findings, herein, the nitrogen-doped carbon encapsulation of Fe composites is investigated to tune the surface active components and introduce defects into the structure. Effects of Li, Mg, Al, Ti, Co, or Ni codoping in these nitrogen-doped carbon-supported Fe spinel composites also aim to improve their electronic properties, besides modulating the selectivity toward high-value-added chemicals in the FTS reaction.

The strategies consist of nitrogen heteroatom introduction into the carbon skeleton to have more defective sites, in addition to regulating the surface properties to improve the selectivity to the products by including the promoters within the Fe species encapsulated in the carbon matrix. This would affect the metal–carbon interactions in carbon-supported catalysts with functional groups and, therefore, facilitate the formation of iron carbides, e.g., Hägg iron carbide (χ-Fe_5_C_2_), cementite (θ-Fe_3_C), hexagonal iron carbides (ε-Fe_2_C and ε-Fe_2.2_C), and Eckström-Adcock carbide (Fe_7_C_3_), which is regarded as the active phase for the FTS reaction.^25,26^

Furthermore, small iron particles bound to nitrogen-doped graphitic functionalized carbon surface could significantly contribute to iron carburization rather than sintering and hence enhance overall catalytic performance, as observed in previous studies.^20,23^

## 2 Experimental Section

### 2.1 Synthesis of the Composites by Polymer-Assisted Combustion with Urea

The Fe_*x*_NC/MFe_2_O_3_ samples were synthesized by the polymer-assisted combustion method, based on the literature reports, with modifications.^27,28^ Typically, 20 g of polyvinylpyrrolidone (PVP, average molecular weight of 40.000, Sigma-Aldrich) was dispersed in 18 mL of isopropanol, under vigorous stirring for 10 min at room temperature. Thereafter, a 0.010 mol·L^–1^ iron nitrate nonahydrate (Fe­(NO_3_)_3_·9H_2_O, Sigma-Aldrich, ≥98%) solution and 0.002 mol·L^–1^ aluminum nitrate nonahydrate (Al­(NO_3_)_3_·9H_2_O, Sigma-Aldrich, ≥98%) were added to the previous solution while stirring for 30 min. Then, a 0.036 mol·L^–1^ urea solution (NH_2_CONH_2_, Sigma-Aldrich, ≥99%) was added to the previous mixture while stirring for 1 h. Subsequently, the mixture was mechanically stirred to obtain a viscous brown solution at a pH = 10, which was heated at 100 °C in an oven for 24 h. After this period, a precipitate was recovered by centrifugation and washed repeatedly with deionized water and ethanol by centrifugation at least six times. Afterward, the product was dried at 100 °C overnight, with the subsequent calcination in an inert atmosphere at 700 °C for 2 h with a heating rate of 1 °C·min^–1^ to obtain the Fe_*x*_NC/AlFe_2_O_3_ composite. The sample was designed as Fe_*x*_NC/AlFe, when aluminum was introduced in the composite as the promoter.

Similarly, other samples designed generically, such as Fe_*x*_NC/MFe_2_O_3_ (M = Ni, Li, Mg, Co, or Ti), were synthesized by following the aforementioned protocol. In these syntheses, instead of aluminum nitrate, nickel, lithium, magnesium, cobalt, and titanium nitrate precursors were added consecutively with iron nitrate with the following notation: Fe_*x*_NC/NiFe, Fe_*x*_NC/LiFe, Fe_*x*_NC/MgFe, Fe_*x*_NC/CoFe, and Fe_*x*_NC/TiFe. A schematic representation of the synthesis of the solids is shown in Figure 2.

**Figure 2:**
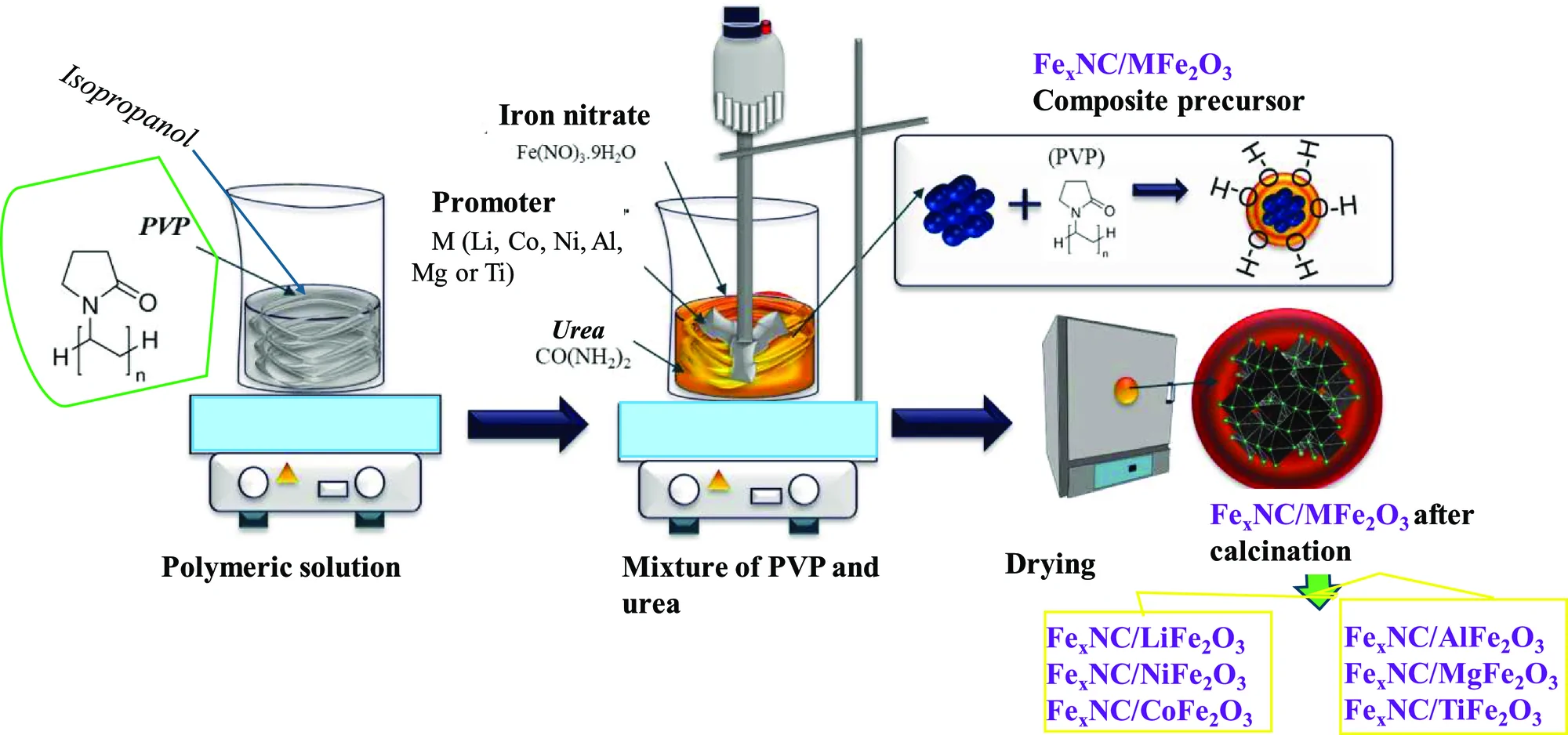
Schematic representation of the polymer-assisted combustion synthesis with urea.

### 2.2 Characterizations

The crystal structure of the catalysts was determined by X-ray diffraction (XRD) in a Bruker model D8 Advance X-ray diffractometer. Cu Kα radiation was used with a voltage of 40 mA and 40 kV. XRD patterns were recorded in the 2θ range of 10–70° using a step size of 0.02° and a step time of 1 s. The XRD patterns were compared to those of the Joint Committee of Powder Diffraction Standards (JCPDS). The Debye–Scherrer equation was used to obtain the crystallite sizes based on the 2θ = 35° (311) plane.

The morphology and elemental composition of the samples were obtained by scanning electron microscopy (SEM) coupled with energy-dispersive X-ray spectroscopy (EDS) using TESCAN VEGA3 equipment. The results were obtained at a 2 kV acceleration voltage. Before analyses, samples were sprayed with a coating of conductive Ag, and the SEM-EDS analyses were conducted.

The textural properties of the solids were examined through nitrogen physisorption analyses using a Micrometrics instrument at −196 °C. The samples were degassed at 250 °C for 2 h in a vacuum, before the analyses.

Raman spectroscopy measurements were obtained in a LabRam spectrometer coupled to a nitrogen-cooled charge-coupled-device detector. The Raman spectra were acquired using a laser power of 10 mW at 532 nm exciting radiation with He–Ne laser. The spectral range was 100–3500 cm^–1^, with 10 data acquisitions per 10 s, with a spectral resolution of 4 cm^–1^.

Mössbauer spectroscopy measurements were obtained on a Wissel spectrometer with transmission geometry. The spectra were recorded for fresh samples at room temperature using a ^57^Co-in-Rh matrix source at 50 mCi. The α-Fe foil was used for calibrations, and the spectra were fitted with a Normos95 software. The isomer shifts have high values owing to the second-order Doppler contribution.

The X-ray photoelectron spectroscopy (XPS) analysis was used to determine the surface composition and the valence state of the samples. The analyses were performed in a Physical Electronics VersaProbe II Scanning XPS photoelectron spectrometer under an ultrahigh vacuum using a monochromatic X-ray Al Kα beam source. The C 1*s* peak at 284.8 eV was used as a reference to calibrate the binding energies.

The Fourier transform infrared (FTIR) spectra of the samples were recorded using a Vertex70 Bruker spectrometer. The spectra were recorded in the 4000–500 cm^–1^ range with a resolution of 4 cm^–1^. Previously, the samples were diluted with KBr in a ratio of 1:1000 for the sample to KBr. For spent catalyst characterizations, attenuated total reflectance Fourier transform infrared (ATR-FTIR) spectroscopy was employed without using KBr.

The transmission electron microscopy (TEM) images of selected samples were obtained with an FEI Tecnai microscope operating at 200 kV. Samples were dispersed in ethanol and deposited onto carbon film-coated copper grids, prior to the analyses.

The reduction behavior of the solids was determined by temperature-programmed reduction (H_2_-TPR) analysis. About 100 mg of the catalysts were placed in a U-shaped quartz tube and preheated at 300 °C for 1 h under Ar flow. After cooling to room temperature, a 10% H_2_/Ar mixture with a flow of 30 mL min^–1^ was introduced into the tube and heated at a rate of 10 °C min^–1^ from room temperature to 1000 °C.

### 2.3 FTS Reaction

The Fischer–Tropsch Synthesis reaction was carried out in a semi-batch high-pressure autoclave reactor from Parr Instruments model 4571. About 2 g of the solids were suspended in 150 mL of *n*-hexadecane and pre-reduced in a hydrogen atmosphere with a flow of 400 cm^3^·min^–1^. Then, the reactor was heated at a rate of 1 °C min^–1^ to 500 °C for at least 2 h. *n*-Hexadecane is an inert solvent under the reaction conditions, having a dissolving capability to decrease the wax layer thickness and extract α-olefin into the mixture.^29^ The catalytic evaluations were performed with feeding H_2_ and CO with a ratio of 1:1 with a total flow rate of 250 cm^3^·min^–1^ using a mass flow controller (Alborg GFC-17). The gas hourly space velocity (GHSV) was 13.5l NTP. gcat^–1^.h^–1^. The FTS reaction was carried out at 240 and 270 °C at 20 atm during 6 h of operation, under constant stirring of 500 rpm, as described in previous findings.^15^ Also, the H_2_ to CO ratio of 2:1 was evaluated. All gases were purchased from White Martins with 99.99% purity. The reaction was carried out in the absence of mass-transfer limitations.

Liquid-phase products were analyzed by gas chromatograph (Shimadzu GC-2010 Plus) equipped with a barrier discharge ionization detector (BID). The gas chromatography (GC) analysis was performed using a DB-5 column with helium as the carrier gas at a constant flow rate of 1.5 mL·min^–1^. The samples were injected in splitless mode at 230 °C, and the oven temperature was programmed to start at 80 °C (held for 2 min), ramped at 5 °C·min^–1^ to 120 °C, and finally increased at 10 °C·min^–1^ to 230 °C, where it was held for 2 min. The identification of the compounds was carried out by comparing their retention times with those of standard hydrocarbon mixtures, specifically utilizing a C_6_–C_8_ standard and a C_10_–C_22_ standard. Given the near-universal response of the BID to organic compounds, raw chromatographic peak areas were treated as directly proportional to mass concentration. The relative weight fraction (*W*_n_) of each hydrocarbon chain length within the collected liquid trap was calculated by normalizing the individual peak area (*A*_n_) against the total area of the fraction.

The intrinsic chain-growth probability (α) for each catalytic condition was determined using the logarithmic formulation of the Anderson−Schulz–Flory (ASF) distribution. A linear regression was performed by plotting ln­(*W*_n_/*n*) against the carbon number (*n*). To ensure the calculated α values accurately reflected the fundamental step-growth polymerization kinetics of the catalysts, highly irregular mass spikes, specifically, a consistent selective anomaly observed at C_10_, were treated as shape-selective deviations due to the support constraints and excluded from the baseline linear regression.

Because the isolated C_10_ fraction constituted a substantial deviation from standard FTS kinetics, often representing the majority of the total liquid mass fraction, a mathematical correction was implemented to reconstruct a realistic, full-spectrum product distribution. First, the theoretical weight fraction for C_9_ was calculated using the intrinsic, anomaly-free α value. Next, the empirical mass ratio of C_10_ to C_9_, as observed in the raw BID chromatograms, was applied to correct the C_10_ mass.

Using the renormalized ASF model, the complete theoretical product distribution (C_1_–C_20+_) was extrapolated for each catalyst condition. This permitted quantifying the catalytic output into standard industrial cuts: light gases (C_1_–C_4_), naphtha (C_5_–C_8_), SAF (C_9_–C_14_), diesel (C_15_–C_20_), and heavy waxes (C_21_^+^).

## 3 Results and Discussion

### 3.1 Morphology and Textural Properties

The morphology of the synthesized samples appears as aggregated slabs with uneven surfaces when considering the low-magnification SEM micrograph (left panel, Figure 3a). The polymer-assisted co-precipitation synthesis consists of using PVP as an amphiphilic component, in which the interaction among negatively charged oxygen atoms of the polymer molecule, Fe^3+^, and M^2+^/M^3+^ cations in a solvent medium is through electrostatic attraction.^30^ As a result, the formation of a chelated metal–nitrogen-PVP composite precursor is likely. Hence, the surface-stabilized composite acts as a structural agent to drive solid morphology. During the synthesis, the urea decomposes into ammonium hydroxide and carbon dioxide gas, and its further reaction in the media generates the carbonate ions, in a similar fashion to the findings.^31^ Besides, these carbonate ions in the presence of the aforesaid chelated metal–nitrogen-PVP precursor help to precipitate the species, providing a stable composite precursor. Such a system has adjustable particle dispersion and control of their growth, since PVP also helps as a modifier for modulating the morphology.^31^ Of importance, the rapid precipitation of the nuclei can coarse the particles, resulting in the aforesaid slab-shaped morphology.^32^

**Figure 3:**
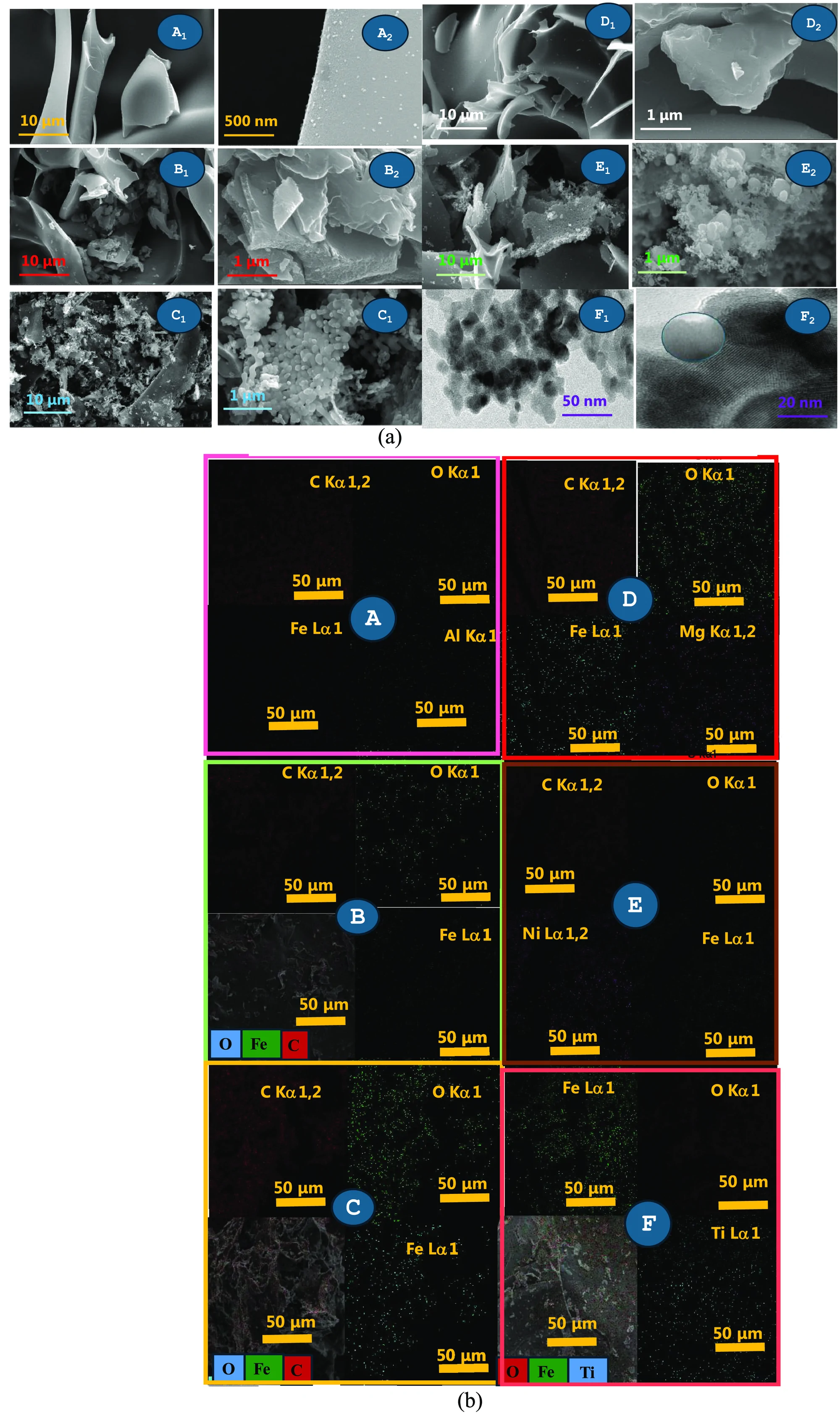
(a) SEM micrographs and (b) EDS analyses of the composites. The letters A, B, C, D and E correspond to the Fe_*x*_NC/AlFe, Fe_*x*_NC/CoFe, Fe_*x*_NC/LiFe, Fe_*x*_NC/MgFe, and Fe_*x*_NC/NiFe, respectively. The high-magnification TEM images for Fe_*x*_NC/MgFe in (a) are included in letter F. The numbers 1 and 2 refer to low- and high-magnification images, respectively. The letter F in (b) is the EDS analysis of Fe_*x*_NC/TiFe.

When the composite is pyrolyzed upon calcination in an inert atmosphere, the PVP combustion is accelerated by urea, giving CO as a reducing agent for the Fe particles. This allows the partial reduction of Fe^3+^ to Fe^2+^ cations of the final composite, as later shown by XRD, XPS, and Mössbauer results. Despite SEM images illustrating that the particle aggregates with each other due to the annealing temperature effects, tunable particle sizes with uniform dispersion are obtained, as further shown by EDS, XRD, and TEM results.

The Fe_*x*_NC/AlFe composite undergoes a smooth slab surface morphology (left panel, SEM image A_1_, Figure 3a) with a more or less uniform shape, in which some particles are visible on it, as shown at higher magnification (left panel, SEM image A_2_, Figure 3a). Indeed, these small particles crystallize on the surface of the large slabs with strong adherence due to the presence of functional groups, as will be shown by FTIR. In addition, Fe_*x*_NC/CoFe shows a morphology that consists of dense superimposed slab platelets, when the solids are imaged at low and higher magnifications (left panel, SEM image B_1,2_, Figure 3a). In the case of Fe_*x*_NC/LiFe, distinct morphologies constituted of well-shaped spheres can be observed (left panel, SEM images C_1,2_, Figure 3a), as an indication of the Li entity influencing the surface morphology of the solids. It is worth noting here that the PVP acts as a template and surfactant, while the urea precursor plays the chelating role of binding the metal during the synthesis. The heating treatment at 700 °C gives the resultant plate morphology, according to the findings.^30^

SEM images of Fe_*x*_NC/MgFe (left panel, SEM images D_1,2_, Figure 3a) display similar morphologies to those of Fe_*x*_NC/CoFe, whereas the particles of Fe_*x*_NC/NiFe (left panel, SEM images E_1,2_, Figure 3a) resemble those of Fe_*x*_NC/LiFe.

The elemental mapping provided by EDS illustrates a uniform distribution of C, Fe, and O on the solid surface of the solids, except for Fe_*x*_NC/NiFe and Fe_*x*_NC/TiFe (right panel, letters E and F in Figure 3b). The Fe/C ratios determined by EDS results are 0.47, 0.34, 0.36, and 0.26 for Fe_*x*_NC/AlFe, Fe_*x*_NC/CoFe, Fe_*x*_NC/MgFe, and Fe_*x*_NC/LiFe, respectively. The amounts of Li and Co on the surface of Fe_*x*_NC/LiFe and Fe_*x*_NC/CoFe are negligible, revealing that these elements could be present in the bulk or leached during the washing of the solids. On the other hand, minor amounts of ca. 2 wt % of Al and Mg are seen for Fe_*x*_NC/AlFe and Fe_*x*_NC/MgFe. In the case of Fe_*x*_NC/NiFe, the Fe/C ratio is 0.95, while carbon is not found by EDS analysis in Fe_*x*_NC/TiFe. This confirms the formation of a bulk structure with the Ti promoter in Fe_*x*_NC/TiFe, whereas a NiFe binary oxide could be formed in Fe_*x*_NC/NiFe, as further shown by the XRD results. Indeed, the amounts of Ni and Ti on the surface of these solids are 13 and 11 wt %, respectively.

TEM image of Fe_*x*_NC/MgFe shows a nanocluster of aggregated particles with an average diameter of 5–30 nm (right panel, letter F in the bottom of Figure 3a). The low-magnification TEM image depicts lattice fringes with a *d* spacing of 0.254 nm, which is associated with the (311) plane of Fe_3_O_4_. It is important to note that magnetite and maghemite have nearly identical spinel structure, which is not distinguishable by TEM alone. Thus, the mixed Fe spinel phase is associated with the presence of both γ-Fe_2_O_3_ and Fe_3_O_4_ phases. This correlates well with XRD results that revealed the existence of such spinel phases, as later shown. A corrugated entity surrounding the surface of the dark particle area seems to be from the encapsulation of the Fe_2_O_3_ spinel phase either in amorphous or graphitized carbon species. Accordingly, the curled wrinkle-like structure is typical of nitrogen-doped carbon with a graphite structure.^33–35^ In agreement, the *d* spacing of 0.325 nm corresponds to the interlayer structural packing of the (002) crystal plane of graphite with diameters close to 25 nm. Besides, the thickness of the layers is estimated to be about 56 nm. Also, some curved fringes are detected, which is suggestive of amorphous carbon formation, as later evidenced by Raman measurements that illustrate the presence of both graphite and amorphous carbon.^36^ This is in line with the findings that show crystalline particle formation during pyrolysis of nitrogen-doped carbon with Fe particles, in which the degree of graphitization of the solids is improved upon amorphous carbon transformation into graphite carbon layers by heating at high temperatures.^33^

The nitrogen adsorption–desorption analyses (Figure 4a) exhibit predominantly type IV isotherms, which are typical of mesoporous materials, in accordance with IUPAC classification.^21^ In the case of Fe_*x*_NC/CoFe, a type IV isotherm with a pronounced H_2_ hysteresis loop at a relative pressure of around 0.45 is indicative of the ink-bottle pores with a wide distribution (Figure 4b). Moreover, interconnected pore networks having considerably larger high surface areas, pore volumes, and pore diameters than the other solids are observed (Table 1).

**Figure 4:**
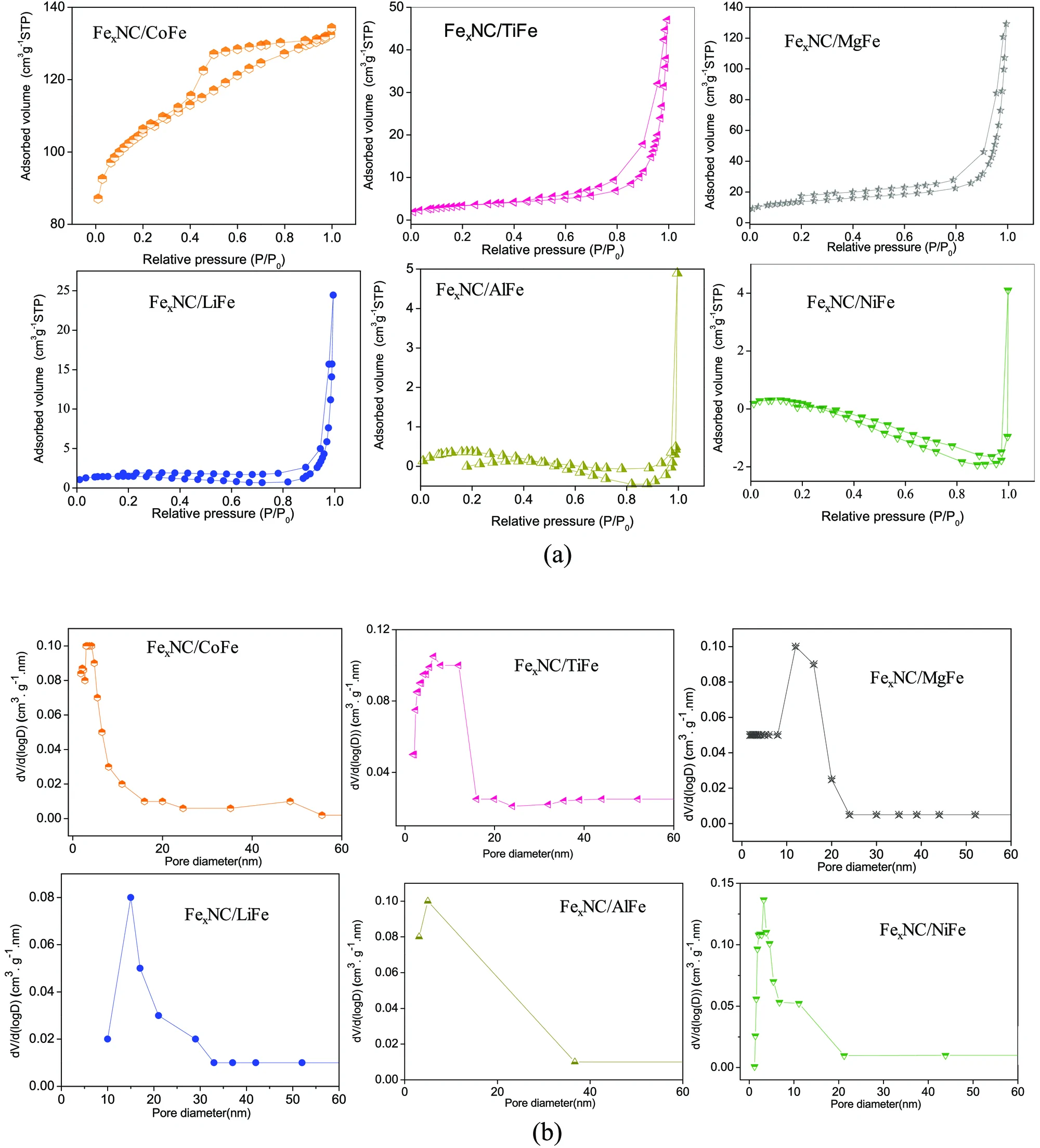
(a) Nitrogen adsorption–desorption isotherms and (b) pore size distribution curves of the composites.

**Table 1:** Textural Properties of the Composites Obtained from the Nitrogen Adsorption–Desorption Isotherms

	surface areas				
sample	S_BET_ ^a^	S_micropore_ ^b^	total pore volume^c^ (cm^3^ g^–1^)	micropore volume (cm^3^ g^–1^)	^b^pore size^d^ (nm)
Fe_*x*_NC/CoFe	358	242	0.11	0.07	3.7
Fe_*x*_NC/TiFe	12		0.07	0.001	2.1
Fe_*x*_NC/MgFe	48	14	0.16	0.05	2.0
Fe_*x*_NC/LiFe		5	0.02	0.002	1.8
Fe_*x*_NC/AlFe		1	0.001	0.001	1.0
Fe_*x*_NC/NiFe		4	0.002	0.002	1.1

a BET surface areas.

b *t*-Plot micropore areas

c The total pore volume was collected from the BJH method.

d Pore diameters were obtained by the adsorption branch of the isotherms.

The well-known oxidation of Co^2+^ to Co^3+^ ions can release carbon dioxide during calcination, leaving behind a larger porosity compared with the other samples. In the case of Fe_*x*_NC/TiFe, Fe_*x*_NC/MgFe, and Fe_*x*_NC/LiFe, the H_3_ hysteresis loop is characteristic of either slit-shaped pores or pore networks with partially filled macropores during capillary condensation.^33^ Moreover, Fe_*x*_NC/TiFe and Fe_*x*_NC/MgFe composites are mesoporous materials regarding their BET surface areas of 5–48 m^2^·g^–1^ and pore diameters above 2.0 nm (Figure 4b), having micropores, whereas Fe_*x*_NC/LiFe has only micropores (Table 1).

Hence, urea interacts with the polymer skeleton to form nitrogen-doped carbon, usually with porous structures, as found elsewhere.^34^ Remarkably, Fe_*x*_NC/TiFe has only mesopores, while Fe_*x*_NC/MgFe and Fe_*x*_NC/LiFe also have micropores.

The Fe_*x*_NC/AlFe and Fe_*x*_NC/NiFe possess type I isotherms (Figure 4a), suggesting microporous materials in these solids. Accordingly, these solids show very low surface areas and pore volumes within the ranges of 1–5 m^2^·g^–1^ and 0.001–0.002 cm^3^·g^–1^ (Table 1). According to the SEM analyses, the solids typically have agglomerated particles as a result of PVP graphitization and urea decomposition into carbonates and gases at relatively high temperatures.

When the gases leave the solids in an inert atmosphere, carbon monoxide is generated during calcination by partially reducing the Fe species and leaving behind a microporous structure with a pore size lower than 2 nm.

### 3.2 Crystal Structure by XRD and Vibrational Properties by Raman and FTIR

The crystal structures of the solids are investigated by XRD patterns. All the diffraction peaks have a relatively low degree of crystallinity (left panel, Figure 5a), except for Fe_*x*_NC/TiFe and Fe_*x*_N/LiFe (right panel, Figure 5b). For Fe_*x*_NC/CoFe, Fe_*x*_NC/NiFe, Fe_*x*_NC/AlFe and Fe_*x*_NC/MgFe, peaks are ascribable to the 2θ = 18.3(111), 30.2 (220), 35.5 (311), 43.2 (400), 53.7 (442), 62.8 (511) and 66.8° (440) crystal planes of the cubic inverse Fe_3_O_4_ spinel phase^38–40^ with space group *Fd*3*m* (JCPDS 8801315). The presence of the cubic γ-Fe_2_O_3_ spinel phase cannot be ruled out, since both maghemite and magnetite inverse spinel structures^39–43^ have XRD peaks in similar positions. Additionally, the broad peak at 25.8° (200) indicates the presence of graphitized carbon with a lattice fringe spacing of ca. 0.251 nm, as found elsewhere.^44^ Of importance, small peaks at the 2θ = 13.3 (100) and 2θ = 26.1° (002) are assigned to the graphitic C_3_N_4_ carbon nitride (JCPDS 871526). According to the findings, C_3_N_4_ carbon nitride decomposes, serving as a precursor to form Fe_*x*_N carbon nitrides, when pyrolysis is conducted at 650–850 °C.^44,45^ As nitrogen and carbon have similar radii, the introduction of the former into the carbon lattice may form chemical nitrogen species and stable nitrogen bonded to the carbon species, e.g., pyrrolic nitrogen, pyridic nitrogen, graphitized nitrogen, and pyridine oxide.^46^ Consistent with these results, the Fe species are polymerized during the synthesis and further calcined at 700 °C to obtain the stable spinel phases and nitrogen-doped Fe carbides with a high degree of graphitization, after pyrolysis.

**Figure 5:**
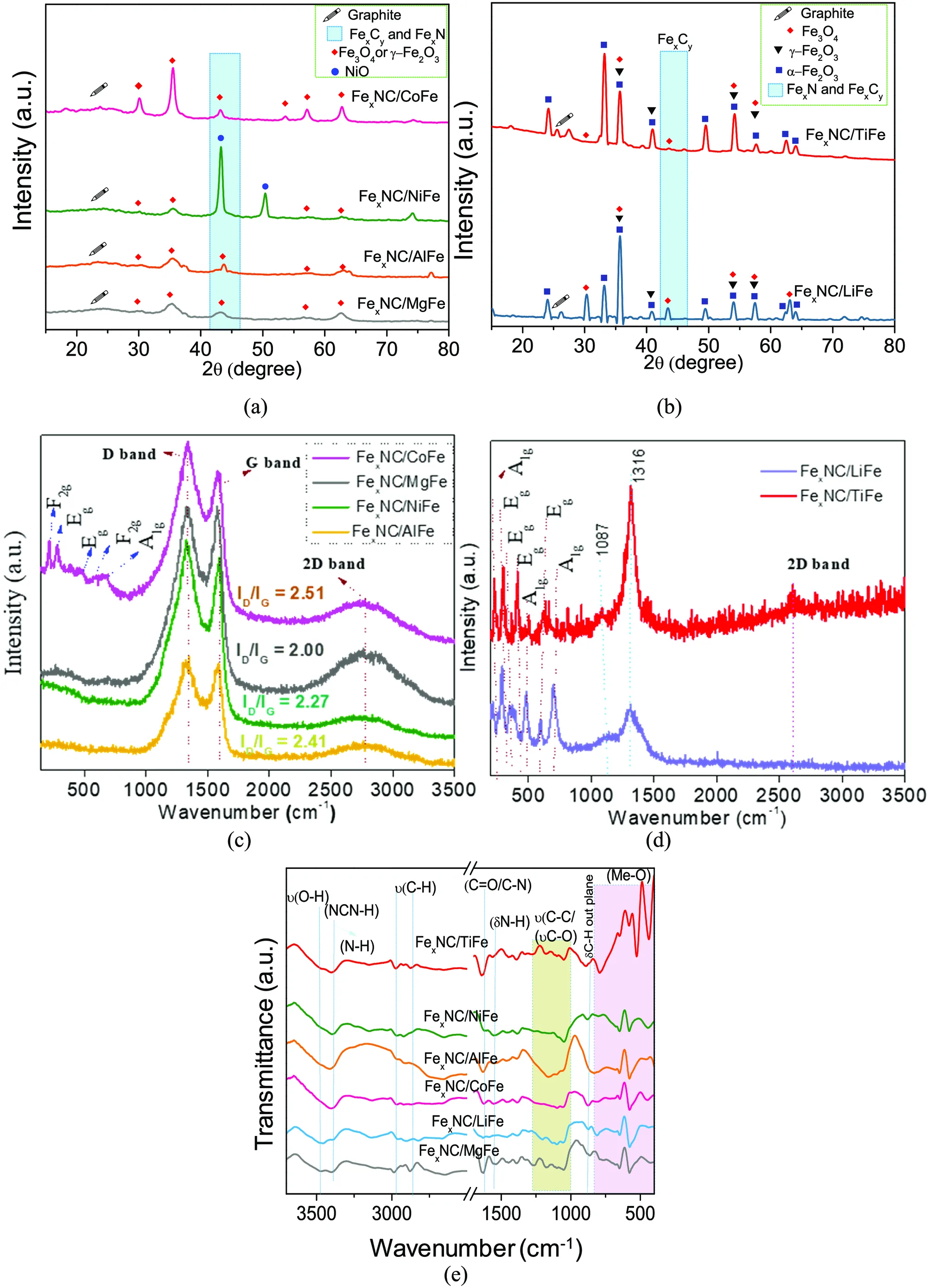
Structural features of the composites: (a) XRD patterns of the semicrystalline solids, (b) XRD patterns of Fe_*x*_NC/TiFe and Fe_*x*_N/LiFe composites, (c) Raman spectra of the semicrystalline solids, (d) Raman spectra of Fe_*x*_NC/TiFe and Fe_*x*_N/LiFe composites, and (e) FTIR measurements of all solids.

Later, XPS results show the existence of surface nitrogen-containing compounds over all solids, with Fe_*x*_NC/TiFe being an exception. Furthermore, the peaks are in similar positions as those of the Hägg (χ-Fe_5_C_2_) and cementite (θ-Fe_3_C) iron carbides (JCPDS 89-6158 and 89-2867), in addition to the Fe_3_N (JCPDS 49-1663), in agreement with the findings.^42,46^

The presence of these iron carbide and iron nitride phases is further confirmed by Raman measurements. Moreover, the lattice parameters of magnetite are *a, b*, and *c =* 8.36984 Å. A small shift in the (311) reflection peak could be caused by variation in the lattice parameters due to the Al^3+^ ion (0.50 Å) substituting a larger octahedral Fe^2+^ site (0.63 Å) or a tetrahedral Fe^3+^ (0.49 Å) one in Fe_3_O_4_ to form a possible AlFe_2_O_4_ spinel structure. Moreover, the slightly higher ions such as Li^+^ (0.60 Å), Mg^2+^ (0.65 Å), Ti^4+^ (0.68 Å), Ni^2+^ (0.69 Å), or Co^2+^ (0.74 Å) impact the solids, causing the expansion of the lattice parameters of Fe_3_O_4_.

This could result in the corresponding MFe_2_O_4_ ferrite formation (M = Li, Al, Ni, Co, Mg, or Ti), although they are not observed probably because their amounts and crystallite sizes are below the limit of detection of XRD. In addition, the bulk content of Fe is ca. 1.0 wt % while that of the M = (Li, Ti, Mg, Co, Al, and Ni) is close to 0.8 wt %, as obtained by chemical analysis.

Concerning Fe_*x*_NC/TiFe and Fe_*x*_NC/LiFe, distinctive diffraction patterns with sharper peaks suggest more crystallized structures. The two samples depict characteristic peaks of the rhombohedral α-Fe_2_O_3_ (JCPDS 84-0311) concomitantly with cubic Fe_3_O_4_ and γ-Fe_2_O_3_ (JCPDS 39-1340) spinel phases. It is also worth noting that the α-Fe_2_O_3_ phase is a result of magnetite oxidation at high temperatures.^47–49^ These peaks appear in similar positions to those of graphite in addition to the iron nitride and iron carbides. Further, the crystallite sizes determined based on the 2θ = 35° (311) for Fe_*x*_NC/CoFe, Fe_*x*_NC/TiFe, and Fe_*x*_NC/LiFe are 18, 25, and 29 nm, respectively. This suggests a small crystallite size of the Fe_2_O_3_ spinel phase for Fe_*x*_NC/CoFe, compared to the other samples.

All of the above observations evidence that the solids are composed of Fe_*x*_C_*y*_ and Fe_*x*_N decorating the surface of a carbonaceous matrix, which encapsulates the Fe_3_O_4_ phase. Particularly, Fe_*x*_NC/TiFe and Fe_*x*_NC/LiFe show the formation of γ-Fe_2_O_3_ and α-Fe_2_O_3_, besides Fe_3_O_4_.

The spectroscopic investigations by Raman and FTIR measurements are used to corroborate the XRD and TEM results. Raman spectroscopy is a helpful technique to elucidate the phase formation in solids through their vibrational properties. At low frequencies, Raman spectrum of Fe_*x*_NC/CoFe (left panel, Figure 5c) displays vibration modes at about 205­(F_2g_), 284 (E_g_), 490 (E_g_), 616 (F_2g_) and 700 cm^–1^ (A_1g_) corresponding to the Raman active phonons modes of the spinel Fe_3_O_4_.^48^ The possibility of the Raman bands at 350 (F_2g_), 510 (E_g_), and 700 cm^–1^ (A_1g_) assigned to the γ-Fe_2_O_3_ cannot be ruled out, since these Raman bands could be superimposed on those of magnetite.^49^ Both magnetite and maghemite crystallize in the inverse and normal spinel structure, belonging to the *Fd*3*m* (*O*_*h*_^7^) space group, while Co_3_O_4_ holds the normal spinel structure.^49^ The low-frequency Raman bands for Fe_*x*_NC/NiFe, Fe_*x*_NC/AlFe, and Fe_*x*_NC/MgFe are not visible owing to the high intensity of the bands at high frequencies. For Fe_*x*_NC/TiFe and Fe_*x*_NC/LiFe (right panel, Figure 5d), Raman bands at low frequency are positioned at about 225 (A_1g_), 247 (E_g_), 292 (E_g_), 411 (E_g_), 496 (A_1g_), 610 (E_g_), and 680 (A_1g_) cm^–1^. This confirms the rhombohedral α-Fe_2_O_3_ structure (*R*3*c* (*D*_6*d*_^3^) space group) with seven phonon lines, in addition to a small shoulder at 1080 cm^–1^ and a broad and intense band at 1316 cm^–1^. Such an intense band is attributed to the doubling of the 2-Longitudinal optical phonon frequency from the interaction of two magnons created close at parallel spin sites.^48^ The shoulder at about 1180 cm^–1^ is due to the CH_2_ modes of the functionalized carbon species.^46,48^

At high frequencies, two Raman bands located at around 1340 and 1580 cm^–1^ (right panel, Figure 5c) correspond to the well-documented D and G bands arising from graphite.^43,46^ The D band is assigned to the disorder-induced carbon owing to the breathing vibration of *sp* ^2^ defective domains in carbon, i.e., glassy carbon or pyrolytic graphite.^50^ The G band is associated with the Raman-allowed E_2g_ symmetric mode in the first-order allowed tangential in-plane stretching of the graphite lattice owing to the perfect stretching vibration of *sp* ^2^-hybridization of ordered carbon.^24,35,41^ Remarkably, the band at around 1500 cm^–1^ is in a similar position to that ascribable to the C_*x*_N_*y*_ carbon nitride and iron carbides.^50–52^ As the *I*_D_/*I*_G_ ratio illustrates either degree of graphitization or structural disorder of carbon materials, the *I*_D_/*I*_G_ ratios of 2.51, 2.41, and 2.27 for Fe_*x*_NC/CoFe, Fe_*x*_NC/NiFe, and Fe_*x*_NC/AlFe are indicative of the largest ratios with the high degree of defects. The *I*_D_/*I*_G_ value for Fe_*x*_NC/MgFe is ca. 2.00, suggesting a lower degree of defective carbon matrix among the solids studied. Notably, it is documented that the iron carbides, iron nitrides, and C_*x*_N_*y*_ possess point intrinsic defects, extended defects (*e.g*., edges, kinks), corners, and/or termination sites for Fe_3_O_4_ interaction.^51,52^ Raman spectra at high frequencies have a band at around 2760 cm^–1^ corresponding to the 2D band for all solids, except Fe_*x*_NC/LiFe (Figure 5d).

FTIR spectra of all solids (Figure 5e) depict a broad absorption band at around 3400 cm^–1^ associated with hydroxyl group stretching vibrations.^49^ The findings also state that the primary (−NH) and secondary (NH) CN–H bonds vibrations are visible through adsorption bands at about 3400 cm^–1^.^43–45^ Moreover, the C–H stretching vibrations at around 2820 cm^–1^ in hydrocarbons, along with C=C from aromatic rings, C=O/C–N bond stretching vibrations from and N–H bending vibrations from heterocyclic compounds are observed at about 1700–1500 cm^–1^.^46^ Besides, the C–C and C–O stretching in carboxylic acid, alcohol, esters, and ethers are seen in the 1300–1000 cm^–1^ range. Furthermore, the Me-O vibrations, mainly the Fe–O bond stretching vibrations, are positioned at around 800 cm^–1^.^39^ The C–H bending of aromatics is located at around 789 cm^–1^. The presence of these absorption bands confirms the XRD assumptions for the presence of the C–N, C=C, −NH_2_, C–O, and C_*x*_H_*y*_O_*z*_ termination and structural defects functional groups bonded to the carbon surface to interact with the Fe species. The formation of the nitrogen-doped carbon supported on the iron species is confirmed by FTIR, Raman spectroscopy, and XRD results.

### 3.3 Electronic States and Surface Composition by Mössbauer and XPS

^57^Fe Mössbauer spectroscopy is used to investigate the valence state of the Fe species, as well as their amounts in the fresh samples (Figure 6a). The Mössbauer spectra of the fresh solids presented in Figure 6a are recorded at −143 °C. We have also recorded the Mössbauer spectra at room temperature, e.g., 27 °C, that show a strong presence of superparamagnetic components due to Fe species in small crystallites (not shown). The Mössbauer spectra of Fe_*x*_NC/LiFe, Fe_*x*_NC/TiFe, and Fe_*x*_NC/CoFe display two well-developed sextuplets each, which evidence the presence of a thermally blocked magnetic phase, i.e., γ-Fe_2_O_3_. The above-mentioned results correlate well with the fact that maghemite is an inverse spinel structure with cation vacancies holding an ordered distribution with tetragonal symmetry and a partially ordered cubic superstructure, or yet, a random distribution (space group *Fd*3*m*).^44^ Besides maghemite, the spectra suggest the presence of nonstoichiometric Fe_3_O_4_ and other spinel phases containing Fe_*x*_NC/CoFe, Fe_*x*_NC/TiFe, and Fe_*x*_NC/LiFe.

**Figure 6:**
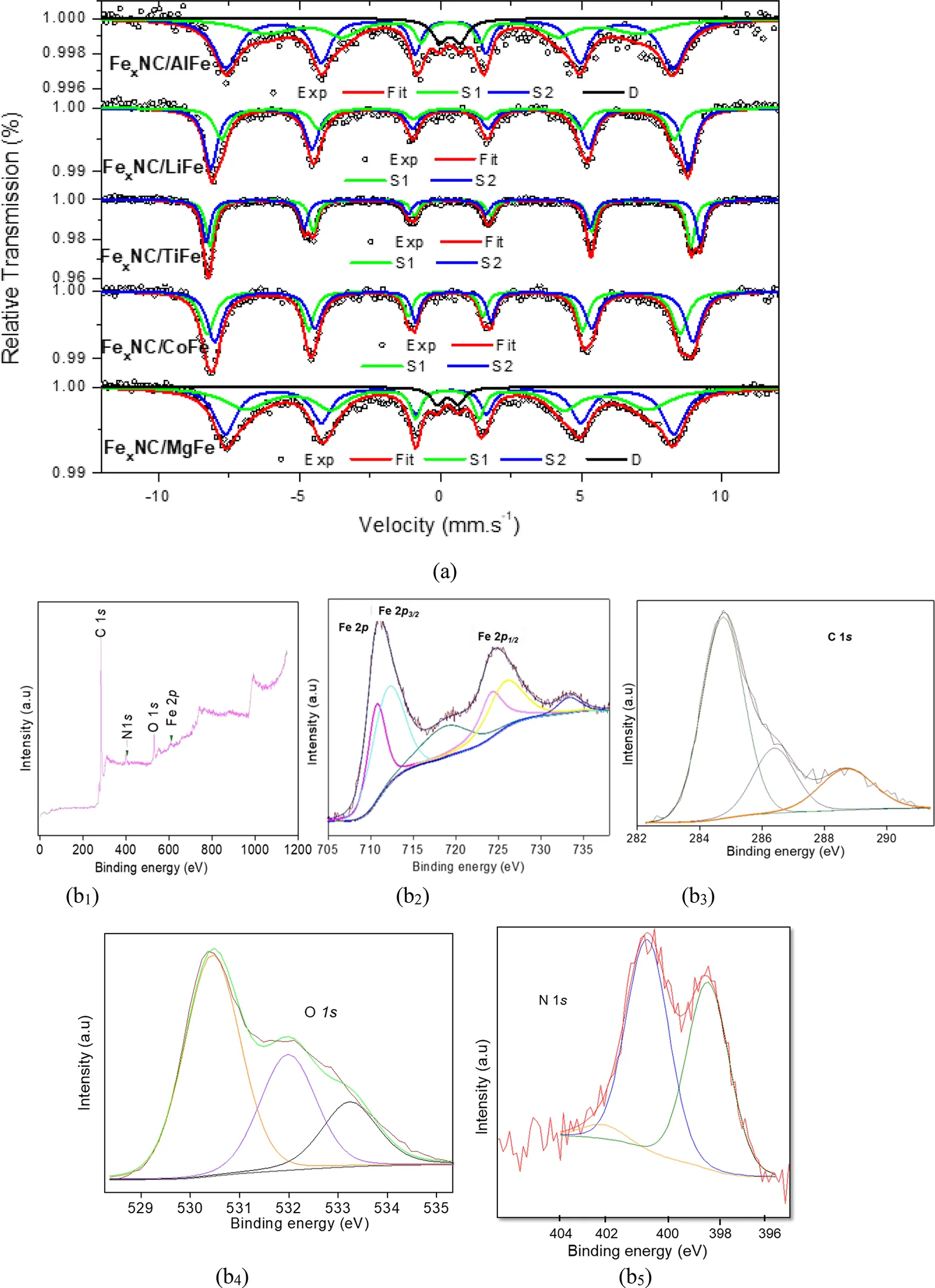
(a) Mössbauer spectra of the solids in study. The symbols S1, S2, and D represent the A tetrahedral site, B octahedral site, and doublet, respectively. The representative XPS spectra of the composites as in the case of Fe_*x*_NC/Fe, where symbols b_1_, b_2_, b_3_, b_4_, and b_5_ correspond to the survey, spectrum, Fe 2*p*, C 1*s*, O 1*s*, and N 1*s* core levels, respectively.

The hyperfine parameters obtained from the deconvolution of the Mössbauer spectra, for instance, isomer shift (δ), quadrupole shift (Q_s_), and hyperfine splitting (Bhf), are listed in Table 2. It is important to note that the spectra are fitted considering a peak width ranging from 0.3 to 0.45 mm·s^–1^.

**Table 2:** Hyperfine Parameters Obtained from the Deconvolution of the Mössbauer Spectra Assigned as Isomer Shift (δ), Quadrupole Splitting (Q_s_), and Hyperfine Splitting (B_hf_)­^a^

composite		Fe_*x*_NC/AlFe	Fe_*x*_NC/LiFe	Fe_*x*_NC/TiFe	Fe_*x*_NC/CoFe	Fe_*x*_NC/MgFe
S1 sextet	δ (mm·s^–1^)	0.41	0.42	0.46	0.27	0.36
A tetrahedral site	Q_S_ (mm·s^–1^)	0.06	–0.06	0.21	–0.07	–0.01
	B_hf_ (T)	40.9	49.8	54.3	52.0	48.6
	area (%)	36.5	36.4	47.3	45.9	45.8
S2 sextet	δ (mm·s^–1^)	045	0.46	0.51	0.57	0.47
B octahedral sites	Q_S_ (mm·s^–1^)	–0.04	–0.03	–0.07	–0.02	–0.05
	B_hf_ (T)	49.1	52.5	52.9	52.6	49.3
	area (%)	56.1	63.6	52.7	54.1	49.2
D doublet	δ (mm·s^–1^)	0.46				0.38
SPM	Q (mm·s^–1^)	0.73				0.76
	area (%)	7.4				5.0

a The term SPM means superparamagnetism feature of the solid.

The core magnetic moments show internal disorder with the hyperfine field values for Fe species in the tetrahedral (A) and octahedral (B) sites.^40^ The Fe^3+^ ions have a smaller isomeric shift value at tetrahedral sites (A-site), when compared with the one at octahedral (B-site) sites. The sextets having the hyperfine field of 49.5–54.3 T and smaller isomer shift are assigned to Fe^3+^ ions at tetrahedral (A) sites (Table 2). The other sextet possesses a hyperfine field of 52.5–52.9 T and larger isomer shifts, which is ascribable to the presence of Fe^3+^ and Fe^2+^ species at the octahedral (B) sites.

In addition, the areas of the Fe species in octahedral (B) sites are higher than those of the tetrahedral (A), indicating that the distribution of the internal magnetic field of Fe^3+^ species in the B sites is predominant in the case of Fe_*x*_NC/LiFe, Fe_*x*_NC/TiFe, and Fe_*x*_NC/CoFe. The area ratios between (B-site) and (A-site) for Fe_*x*_NC/AlFe, Fe_*x*_NC/LiFe, Fe_*x*_NC/TiFe, Fe_*x*_NC/CoFe, and Fe_*x*_NC/MgFe are 1.53, 1.74, 1.11, and 1.08, respectively.

These values indicate higher Fe occupancy at B sites along with the presence of a certain amount of a second element occupying the B-site in Fe_*x*_NC/AlFe and Fe_*x*_NC/LiFe. Also, the spinel phases containing Al, Ti, Co, or Mg cannot be ruled out. It is worth mentioning that Fe_3_O_4_, having a nonstoichiometric structure with cation vacancies at the octahedral site, is also present, with the sextets and magnetic hyperfine splitting parameters having similar values, in accordance with the findings.^37,39,40^

All other hyperfine parameters, along with the superparamagnetic relaxation of fine particles, align with the XRD and Raman spectroscopy results that demonstrate the existence of magnetite in these samples. In particular, the presence of CoFe_2_O_4_ in Fe_*x*_NC/CoFe is confirmed considering the hyperfine splitting close to 50 T.^40^ Thus, a possible mixture of both Fe_3_O_4_ and γ-Fe_2_O_3_ in Fe_*x*_NC/LiFe, Fe_*x*_NC/TiFe, and Fe_*x*_NC/CoFe is likely owing to the high temperature of pyrolysis to obtain the composites. Mössbauer spectra of Fe_*x*_NC/AlFe and Fe_*x*_NC/MgFe are deconvoluted into two sextets and one central doublet. The pyrolysis temperature at 700 °C is enough to oxidize these iron oxides into crystalline hematite α-Fe_2_O_3_ with a magnetic hyperfine field of ca. 40.9–49.3 T, a quadrupole splitting from −0.01 to 0.06 mm·s^–1^, and an isomer shift in the 0.38–0.47 mm s^–1^ range. These features match well with those of superparamagnetic Fe^3+^ and Fe^2+^ ions in both Fe_3_O_4_ and γ-Fe_2_O_3_, in addition to the superparamagnetic Fe^3+^ entity present in α-Fe_2_O_3_.^41,44^

Moreover, the existence of Fe_*x*_N iron nitrite and iron carbides, i.e., Fe_3_C (*Pnnm* space group) and χ-Fe_2_C_5_ (*Pnnm* space group) with similar structures (*Pbcn* space group), cannot be ruled out, since literature reports found Mössbauer parameters with similar values.^33,38^ Thus, the magnetite phase is synergistically embedded in the Fe_*x*_N–C carbon matrix, according to Raman, XRD, and Mössbauer measurements.

XPS analyses are performed to investigate the chemical state and surface composition of the composites. The survey XPS spectra of the bare Fe_*x*_NC/Fe (Figure 6b_1_) depict C, N, Fe, and O elements on the solid surface. This confirms the assumptions of either Fe–N or Fe–C bonds on solid surfaces owing to the successful doping of nitrogen into iron nitrides and iron carbides with carbon matrices. For all the Fe 2*p* core levels (Figure 6b_2_), two asymmetric peaks appear as a spin–orbit doublet centered at around 710 and 724 eV, corresponding to Fe 2*p*_3/2_ and Fe 2*p*_1/2_, respectively (Table 3). These peaks are accompanied by satellites at around 710 eV, for which the lowest binding energy is ascribed to the Fe^2+^ species.^45–47^ In the case of Fe^3+^ octahedral species, the binding energy is reported at 710.8 eV, while the Fe^3+^ tetrahedral species has a peak at 713.3 eV, all of them from magnetite.^42,47^

**Table 3:** Binding Energy of C 1*s*, O 1*s*, Fe 2*p*, N 1*s*, Al 2*p*, Co 2*p*, Ni 2*p*, Ti 2*p*, Mg 1*s*, and Li 1*s* Core Levels

composite	C 1*s*	O 1*s*	Fe 2*p*	N 1*s*	Al 2*p*	Co 2*p*	Ni 2*p*	Ti 2*p*	Mg 2*s*	Li 1*s*
Fe_*x*_NC/AlFe	284.8	530.5	710.8	398.5	74.0					
	286.6	532.1	724.1	400.4						
	288.8	533.7		402.4						
Fe_*x*_NC/CoFe	284.8	530.3	710.7	398.5		780.0				
	286.6	532.3	724.3	400.7		795.5				
	289.0	533.8		402.4						
Fe_*x*_NC/LiFe	284.8	530.6	711.0	398.3						55.4
	286.6	532.5	724.6	400.6						56.2
	288.9	533.9		402.1						
Fe_*x*_NC/MgFe	284.8	530.4	710.6	398.5					88.6	
	286.6	532.3	724.2	400.7						
	288.9	533.8		403.4						
Fe_*x*_NC/NiFe	284.8	530.3	710.8	398.7			873.2			
	286.6	531.9	724.3	400.4			855.4			
	288.7	533.5								
Fe_*x*_NC/TiFe	284.7	530.7	710.9					458.5		
	286.3	531.6	724.6					464.4		
	288.5									

Moreover, another shakeup peak at about 718.1 eV indicates the coexistence of Fe^2+^ and Fe^3+^ ions represented by either Fe_3_O_4_ or γ-Fe_2_O_3_.^42,48^ This is consistent with the XRD, Raman, and Mössbauer measurements. Additionally, the Fe 2*p* positions for Fe_*x*_NC/TiFe and Fe_*x*_NC/LiFe shift toward higher binding energies relative to the other samples, which can indicate the incorporation of Co and Ti ions into the ferrite lattice. Another assumption is that the appearance of the satellite peak at 719 eV along with the Fe 2*p*_3/2_ core level at 710.7 eV is assigned to the Fe^3+^ ions that exist in the γ-Fe_2_O_3_ and α-Fe_2_O_3_ for Fe_*x*_NC/TiFe and Fe_*x*_NC/LiFe. These results fully agree with the findings that relate these features to the existence of Fe^3+^ ions for both the γ-Fe_2_O_3_ and α-Fe_2_O_3_ particles.^47,48^ The C 1*s* core levels (Figure 6b_3_) are deconvoluted into three peaks, with the most intense at around 284.6 eV owing to the adventitious carbon/C–C bonds.^34,40^ Furthermore, the peaks at around 286.3 and 288.2 eV correspond to C–N/C–O/C=O and *sp* ^3^ hybrid carbon atoms/carbonate.^48^

Notably, chemical entity signals are likely due to the carbonaceous layer around the Fe–C or Fe–N bonds, as previously shown by Raman and FTIR results. Moreover, the Fe/C ratios are very low in all solids, which is indicative of the Fe entities mainly on the bulk, with that of Fe_*x*_NC/TiFe of ca. 0.04 (Table 4), confirming the presence of Fe on the surface of the solid.

**Table 4:** Fe/C, N/Fe, N/C, M/C, and M/Fe Obtained from XPS Results^a^

sample	Fe/C	N/Fe	N/C	M/C	M/Fe
Fe_*x*_NC/LiFe	0.010	3.88	0.04	0.170	14.3
Fe_*x*_NC/AlFe	0.004	15.90	0.06	0.005	1.33
Fe_*x*_NC/TiFe	0.004			0.412	0.98
Fe_*x*_NC/MgFe	0.006	8.26	0.05	0.003	0.47
Fe_*x*_NC/NiFe	0.010	2.85	0.04	0.008	0.54
Fe_*x*_NC/CoFe	0.004	11.70	0.05	0.003	0.67

a M indicates the Al, Co, Ni, Mg, Li, and Ti elements.

For O 1*s* core levels (Figure 6b_4_), three contributions at 530.3, 531.4, and 533.4 eV are found with respect to lattice oxygen, oxygen vacancies/hydroxyl groups, and carbonates, respectively.^46^ It is noteworthy that the O^2–^ lattice arises from the surface Me–O bonds from the metal oxides, mainly Fe–O, holding the major peak areas with the minor contributions from hydroxide and carbonate species and oxygen vacancies. The Fe_*x*_NC/TiFe has only two O 1*s* core level peaks at 530.7 eV being characteristic of the lattice oxygen of the spinel oxides, and another peak at around 531.6 eV assigned to the physisorbed OH groups.

The XPS spectrum of N 1*s* core levels (Figure 6b_5_) is deconvoluted into three contributions at 398.4/399.3, 400.9, and 403.3 eV, which are consistent with *sp* ^2^ hybridization (C–N=C), pyrrolic nitrogen, graphitic nitrogen, and the amino functional group, respectively.^27,49^ Moreover, bulk carbon, nitrogen, and oxygen contents determined by elemental analysis of all solids are approximately 82.0, 6.5, and 9.7 wt %, respectively. The presence of these nitrogen-based species reveals their accumulation on solid surfaces in the form of C–N bonds, confirming the existence of carbon nitride on the surface of the solids with N/C ratios between 0.04 and 0.06 (Table 4). Also, the N/Fe ratios suggest the possibility of a strong electronic interaction between Fe entities and the neighboring nitrogen-doped carbons. This correlates with the formation of Fe nitrides as Fe_*x*_N in the carbonaceous matrix, which encapsulate Fe_3_O_4_, as found elsewhere.^50,51^

The Fe_*x*_NC/TiFe is an exception, since no obvious N 1*s* peaks are found on the surface, suggesting Fe_3_O_4_ or α-Fe_2_O_3_ is supported on the carbonaceous matrix. To sum up, the aforementioned results for C 1*s*, N 1*s*, O 1*s*, and Fe 2*p* core levels provide additional evidence of the PVP and urea action in stabilizing and reducing the Fe species partially to bind them on carbon surfaces. Further, pyrolysis of the solids gives a doping of the carbonaceous matrix with nitrogen. Thus, these results confirm the FTIR and Raman spectroscopy, and Mössbauer results that suggested that nitrogen doping sites on the carbon support exist as C–N bonds while strong Fe–N and Fe–C interactions can be achieved.

In addition, the surface Fe^2+^/Fe^3+^ ratio is found to be 0.77–0.80 for Fe_*x*_NC/MgFe, Fe_*x*_NC/AlFe, and Fe_*x*_NC/NiFe, which is greater than the obtained value of 0.5 for stoichiometric Fe_3_O_4_. This reveals that these solids have a more defective structure, in contrast to the Fe_*x*_NC/CoFe and Fe_*x*_NC/TiFe. A full XPS spectrum scan of the solids depicts the characteristic peaks for the Co 2*p*, Ti 2*p*, Ni 2*p*, Mg 1*s*, Li 1*s*, and Al 2*p* core levels. In the case of the Co 2*p* core level, binding energies at 873.2 and 855.4 eV for Co 2*p*_3/2_-Co 2*p*_1/2_ doublets correspond to Co^3+^/Co^2+^ entities, which is suggestive of CoFe_*x*_O_4_ (Fe/Co ratio of 0.67 in Table 4) or Co_3_O_4_ or on a solid surface, as found elsewhere.^49,53^

The obtained Mg/Fe atomic ratio of 0.47 is assignable to the Mg^2+^ ions diffusing through octahedral and tetrahedral sites of Fe_3_O_4_ to form the MgFe_2_O_4_ structure finely dispersed on the carbon surface, in line with the findings.^49^ For Ti 2*p* core levels, the main peaks are at about 458.5 and 464.4 eV, assigning the Ti 2*p*_1/2_ and Ti 2*p*_3/2_ doublets for Ti^3+^ entities interacting with Fe^2+^/Fe^3+^ from α-Fe_2_O_3_ or γ-Fe_2_O_3_, as in the case of nitrogen-doped carbon.^53^ The Ti/Fe ratio of 0.98 in Table 4 confirms these assumptions. For the Ni 2*p* core level, the peaks at around 780.0 and 795.5 eV are attributed to the Ni 2*p*_3/2_-Ni 2*p*_5/2_ doublet, revealing the Ni^2+^ species (Ni/Fe ratio of 0.57) from surface NiFe_2_O_4_ in nitrogen-doped carbon composites.^54^ The Li 1*s* has peaks at 55.4 and 56.2 for Li^+^ ions, with a large amount detectable on the solid surface, which reveals the presence of Li-nitrogen-doped carbon/Fe_3_O_4_ composite, in agreement with the findings.^7^ The Mg 1*s* possesses a binding energy of ca. 88.6 eV for Mg^2+^ ions, and Al 2*p* has a peak at 74 eV for Al^3+^ species on a solid surface.^34^

### 3.4 Reduction Behavior

The reduction behavior is analyzed by the H_2‑_TPR curves for selected nitrogen–carbon-supported Fe samples. Figure 7 illustrates that Fe_*x*_NC/AlFe and Fe_*x*_NC/TiFe have similar curve shapes, revealing at least three hydrogen consumption maxima. The H_2‑_TPR curve (not shown) for the nitrogen-containing carbon has no reduction peak under the TPR conditions evaluated. This is consistent with literature reports showing that the carbon supports are generally gasified at temperatures above 700 °C to produce methane and thus, only a negative of negative peak is observed.^55^

**Figure 7:**
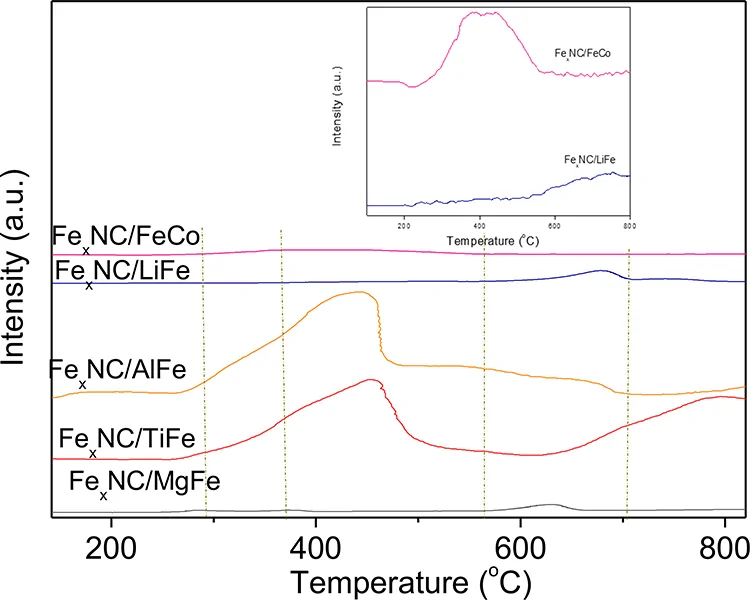
Reducibility of the composites obtained by H_2_-TPR curves. The inset shows the amplified TPR curves of Fe_*x*_NC/CoFe and Fe_*x*_NC/LiFe.

For Fe_*x*_NC/MgFe, two regions of reduction at around 250–350 and 550–650 °C are found. The first peak, located at a low temperature of 295 °C, is due to the reduction of superficial α-Fe_2_O_3_ to Fe_3_O_4_, whereas the second reduction region spanning from 535 to 650 °C is associated with the reduction of the γ-Fe_2_O_3_/Fe_3_O_4_ phase, which is present in all solids to metallic Fe.^12,13,55^ When Ti acts as a promoter in the nitrogen-doped carbon-supported magnetite, the first curve of the TPR reduction peaks displaces to lower temperatures with maxima at 455 °C, whereas the higher-temperature peaks shift to close to 780 °C, indicating that the surface Fe_3_O_4_, iron carbides, iron nitrides, and α-Fe_2_O_3_ are easily reduced owing to either big particles sizes (SEM-EDS) or the weak interaction with the support (Raman, Mössbauer and XPS results).

The Fe_*x*_NC/LiFe is reduced at slightly higher temperatures compared to that of the Ti promoter counterpart (Figure 7, included). In accordance, the addition of alkali promoters, such as lithium, to the Fe-based oxides strengthens the Fe–O bonds by restraining the reduction of the solids.^56,57^ The low intensity of the TPR curve is due to the low amounts of Fe ca. 0.86 wt % and Li of ca. 0.74 wt % in the solids.

On the other hand, Fe_*x*_NC/CoFe has the corresponding Fe_3_O_4_, γ-Fe_2_O_3_, and Co_3_O_4_ surface spinel phase (XPS and XRD results) that may cause the shift of the reduction peaks to higher temperatures, e.g., 200–600 °C, in addition to a second reduction peak above 800 °C, as shown in the amplified Figure 7. Besides, the TPR curve of Fe_*x*_NC/CoFe shows very low-intensity peaks due to the fact that the Co species can be oxidized during the analyses.^47^

It is suggestive of a greater interaction between iron species in the spinel ferrites and carbon matrix. Literature reports show that the reduction of the spinel ferrites is difficult compared to the γ-Fe_2_O_3_ and Fe_3_O_4_ counterparts.^12,58^ Also, it may be due in large part to the presence of smaller Fe particles strongly interacting with the nitrogen-doped graphitized carbon support, as shown by XRD and Mössbauer results.

For the aluminum-promoted composite, the temperature of the first reduction step from maghemite to magnetite is shifted to a slightly higher temperature than the other solids, while the second reduction peak is divided into two additional peaks owing to the reduction of the spinel phase ferrite metallic Fe through the wüstite (FeO) intermediate and its further reduction to FeO.^15^ Besides, it cannot be excluded that the gasification peak is moved to higher temperatures owing to the carbon under the effect of metal species.^7,51^

Thus, the addition of Al in the nitrogen–carbon-supported Fe composite may create a charge imbalance, yielding the formation of oxygen vacancies in the spinel ferrites formed. Upon exposure to the reduction atmosphere, the stabilization of Fe^2+^ in such a kind of structure may inhibit the migration of Fe species to the solid surface, giving less reducible solids compared to the Li- and Ti-containing solids.

### 3.5 Catalytic Performance for the FTS Reaction

The catalytic performances of the composites in the FTS reaction are evaluated under distinct FTS reaction conditions. Analysis of the raw chromatograms by the BID detector reveals a typical Anderson-Schulz–Flory (ASF) chain growth with the exception of the C_10_ fraction. Across nearly all catalytic conditions, regardless of the active metal, temperature, or H_2_:CO ratio, the isolated C_10_ fraction is produced in high disproportion to its neighboring chain lengths. Instead of exhibiting the steady, logarithmic decline in yield predicted by standard step-growth polymerization kinetics, the C_10_ mass frequently constituted between 70 and 90% of the entire liquid trap. This selective behavior strongly suggests that the liquid hydrocarbon phase products consist of chain length product equivalent to the gasoline range. It is worth emphasizing that the distinct promoter added to the Fe_*x*_NC/MFe catalyst does not change the hydrocarbon chain length, since there is no variation in the total proportion of hydrocarbons with chain lengths associated with the gasoline, kerosene, and diesel range. This could be a result of the accumulation of these products in the pores of the solids, wherein growing hydrocarbon chains are sterically hindered from further propagation and are forced to terminate or crack, resulting in a local deviation from the ASF distribution.

Table 5 presents the intrinsic Anderson-Schulz–Flory (ASF) chain-growth probabilities (α) for the evaluated catalysts by varying temperatures and H_2_:CO molar ratios. The resulting coefficients provide a quantitative measure of each catalyst’s propensity for chain propagation versus termination, serving as a critical metric for evaluating their overall viability in synthesizing targeted fuels. As detailed in Table 5, the type of promoter added to the nitrogen-doped carbon-encapsulated Fe composites significantly alters the chain-growth dynamics. While Fe_*x*_NC/CoFe consistently exhibits termination rates as in the typical FTS reaction for liquid fuels, the Fe_*x*_NC/MgFe and Fe_*x*_NC/AlFe have distinct kinetic shifts toward higher mass fractions.

**Table 5:** Intrinsic Anderson-Schulz–Flory (ASF) Chain-Growth Probabilities (α) for the Evaluated Catalysts at Varying Temperatures and H_2_ to CO Molar Ratios

catalyst	temperature (°C)	H_2_-to-CO molar ratio	ASF (α)
Fe_*x*_NC/MgFe	240	1:1	0.70
Fe_*x*_NC/MgFe	270	1:1	0.94
Fe_*x*_NC/MgFe	240	2:1	0.85
Fe_*x*_NC/MgFe	270	2:1	0.73
Fe_*x*_NC/NiFe	270	1:1	0.82
Fe_*x*_NC/NiFe	240	2:1	0.30
Fe_*x*_NC/AlFe	240	1:1	0.94
Fe_*x*_NC/AlFe	270	1:1	0.75
Fe_*x*_NC/AlFe	240	2:1	0.82
Fe_*x*_NC/AlFe	270	2:1	0.88
Fe_*x*_NC/CoFe	270	1:1	0.68
Fe_*x*_NC/CoFe	240	2:1	0.55
Fe_*x*_NC/CoFe	270	2:1	0.59
Fe_*x*_NC/LiFe	270	1:1	0.33
Fe_*x*_NC/LiFe	270	2:1	0.28
Fe_*x*_NC/TiFe	270	1:1	0.53
Fe_*x*_NC/TiFe	240	2:1	0.31

The product selectivity of the catalysts across the entire hydrocarbon spectrum is evaluated based on the intrinsic chain-growth probability (α) as the primary kinetic metric. As described previously, these values are derived from the linear regression of the ASF distribution, excluding the shape-selective C_10_ mass to isolate the fundamental step-growth polymerization kinetics. The resulting parameters reveal that the reaction pathways are governed by a complex interplay associated with the presence of the Fe^2+/^Fe^3+^ entities in Fe_*x*_NC/MFe_2_O_3_ spinel and the iron carbides and nitrides as active species (XRD, Raman and Mössbauer results), promoter added to the composite, and the operating conditions.

The type of promoter added to the nitrogen-doped carbon-encapsulated Fe composites is shown to be the dominant driver of the product distribution. Accordingly, the Fe_*x*_NC/CoFe displays the well-known behavior, yielding the lowest and most stable chain-growth probabilities under all conditions evaluated. Such an effect indicates a relatively fast rate of chain termination compared to the propagation step, resulting in a product profile heavily concentrated in light gases, naphtha, and lighter liquid fractions, with minimal heavy-wax formation. The findings suggest that such FTS reaction conditions increase the autotermination of hydrocarbon chains, which leads to unsaturated hydrocarbon formation, rather than increasing the termination of hydrocarbon chains by means of chain transfer induced by hydrogen.^18,19^

Contrarily, Fe_*x*_NC/MgFe and Fe_*x*_NC/AlFe show distinct kinetics, resulting in significantly higher chain-growth probabilities. An α value exceeding 0.85 indicates a higher propagation rate and highly suppressed termination, biasing the mass balance toward the production of long-chain waxes. The reduced linearity of the ASF distribution is associated with the very low textural properties of the solids, in particular Fe_*x*_NC/AlFe, in turn revealing that mass-transfer limitations within the porous structure of the catalyst are increasing the residence time of intermediate species. This prolonged retention force suggests a possible readsorption of chains and growth into heavier products rather than desorbing as lighter, volatile products.

In the traditional FTS reaction, the activation energy for chain desorption, i.e., termination, is higher than that for propagation; therefore, increasing the reaction temperature theoretically decreases α, shifting the distribution toward lighter fractions. This expected Arrhenius behavior is observed under specific conditions: for example, with increasing temperature from 240 to 270 °C for Fe_*x*_NC/AlFe and Fe_*x*_NC/MgFe at the H_2_:CO of 2:1. On the contrary, Fe_*x*_NC/MgFe shows a chain-growth probability increment from 240 to 270 °C with a H_2_:CO of 1:1. These results, therefore, suggest that at low temperatures, heavy waxes condensed either on surface or within the Fe_*x*_NC/AlFe and Fe_*x*_NC/MgFe pores may sterically blocking active sites and hence, impeding further chain growth. Upon raising the temperature to 270 °C, these waxes likely gained sufficient mobility to clear the pores.

Moreover, Fe_*x*_NC/AlFe and Fe_*x*_NC/MgFe have condition-dependent responses to hydrogen. While the Fe_*x*_NC/MgFe at 270 °C followed the expected trend, e.g., reducing α upon doubling the hydrogen ratio, the Fe_*x*_NC/MgFe shows an increase in chain-growth probability under the same hydrogen-rich conditions. This contradiction suggests that, under specific conditions, the H_2_:CO ratio dictates more than just termination kinetics and might be modulating the oxidation state of the active metal. It is expected that the H_2_:CO ratio of 1:1 undergoes Fe surface partial oxidation, thereby degrading its ability to propagate carbon chains. Elevating the H_2_:CO ratio to 2:1 likely maintains the active Fe in a fully reduced, active state, enabling it to synthesize longer wax chains despite the higher partial pressure of the terminating hydrogen agent.

Adding a Mg promoter to Fe_*x*_NC/MgFe accounts for the inhibitive reduction of hematite to Fe_3_O_4_ or metallic Fe (TPR results). Thus, Mg action as a promoter depicts strong interactions with Fe, with the consequent formation of hardly reducible Fe species and, thus, facilitating the occurrence of both CO hydrogenation and WGS reactions on the Fe_*x*_NC/MgFe. All these factors result in the deactivation of the Fe_*x*_NC/MgFe and Fe_*x*_NC/AlFe by product accumulation on the solids, by increased carburization rates, as further evidenced by the XRD, Raman, and FTIR spent catalyst characterizations.

For Fe_*x*_NC/LiFe, the calculated α values are very low at 270 °C. From an intrinsic kinetic standpoint, the Fe_*x*_NC/LiFe gives primarily methane and light gases at the expense of the chain propagation. Although the Fe_*x*_NC/LiFe has very low textural properties, the light fractions production in the FTS reaction is more influenced by the structural properties and strong electronic interactions among Fe_2_O_3_ spinel nanoparticles, Li species, and carbon matrix. Because the alkali elements, e.g., Li or Mg, exert an action as structural promoters to iron-based catalysts, they could affect the reduction of the Fe-based catalysts, which in turn improves the adsorption and desorption of the syngas reactants on the surface of the catalysts.^57,59^ Besides, TPR results indicate that Li addition to the Fe_*x*_NC/LiFe catalyst results in a strengthened Fe–O–Li bond, providing a strong interaction, which facilitates the reduction of Fe_2_O_3_ to Fe_3_O_4_ and its further reduction to metallic iron. Thus, the presence of Li^+^ ions as an alkali component of the iron oxide is also expected to modify the surface chemistry at the surface of Fe_3_O_4_ supported on nitrogen-doped carbon composite (XPS results) and impact the catalytic performance in the FTS reaction. Further spent solid characterizations reveal that the alkalinity of the Li promoter appears to inhibit carburization of the Fe_*x*_NC/LiFe, with phase transformations being the leading cause of the deactivation of the catalyst.

The Fe_*x*_NC/TiFe exhibits a high sensitivity to the reaction environment. Under mild conditions, e.g., 270 °C, 1:1, the Ti promoter added to the nitrogen-doped carbon-encapsulated Fe composite shows a classical FTS reaction catalyst behavior, maintaining a stable chain-growth probability that targeted the light distillate and gasoline cuts. Because of this propagation baseline, a significant volume of mass reaches the physical pore constraints of the support, resulting in 12.4 wt % of the total product mass being accumulated at C_10_. However, shifting the operating conditions to 240 °C induces a profound kinetic change. In a hydrogen-rich environment, e.g., 240 °C, 2:1, the catalyst’s propagation capability decreases significantly, leading to rapid termination and a shift toward light fractions.

Remarkably, cobalt itself is an active metal in the FTS reaction,^51,60^ but the addition of Co as a promoter to Fe-based catalysts is proven to have a positive effect on the catalytic performance of Fe_*x*_NC/CoFe. As found elsewhere,^61,62^ the bimetallic FeCo catalysts depict a higher reducibility compared to their individual Co_3_O_4_ and Fe_2_O_3_ phases, besides the possibility of forming a FeCo alloy at some compositions and better dispersion of the active phases, all of these factors contributing to the additive FeCo properties for catalytic performance, in agreement with the obtained results.

Because hydrogen acts as the primary terminating agent in the FTS reaction via the hydrogenation of the growing alkyl chain, a higher H_2_:CO ratio is expected to decrease the α value. This principle held true for the classical cobalt catalyst at 270 °C, where increasing the syngas ratio from 1:1 to 2:1 decreases the α value from 0.684 to 0.596, driving the distribution toward lighter gases.

For Fe_*x*_NC/NiFe, a strong dependence on reaction conditions is observed. In a hydrogen-rich environment, e.g., 240 °C, 2:1 H_2_:CO, the Ni addition to the nitrogen-doped carbon-encapsulated Fe composite shows high termination rates, as evidenced by a very low α. However, under lower hydrogen availability and higher temperatures, e.g., 270 °C, 1:1 H_2_:CO, chain propagation is restored, highlighting nickel’s high sensitivity to syngas partial pressures. Additionally, Ni incorporation in Fe-based catalysts remarkably promotes synergistic effects attributed to their electronic and geometric structures.^63^ The conversion of syngas to synthetic fuels over these bimetallic FeNi catalysts is determined by their crystallite surface composition, Fe-oxide particle dispersion, and small crystallite sizes, resulting in increased Fe reduction and carburization rates.^62,63^ Thus, the addition of Ni greatly impacts the activity of Fe_*x*_NC/NiFe through the reduction of Fe particles, by means of a probable Fe–Ni alloy formation affecting further the carburization step, in accordance with the reports.^64^

There is no straight correlation between the morphology and porosity of the solids to ascertain the diffusion limitations and hydrocarbon chain-growth probability in FTS.

As previously mentioned, the C_10_ fraction has a selective anomaly rather than a standard product of step-growth polymerization. Because its formation is likely governed by steric constraints or specific pore geometries rather than pure surface kinetics, the yield of C_10_ is sensitive to variations in the active metal, reaction temperature, and syngas molar ratio. Under optimal conditions, Fe_*x*_NC/NiFe at 270 °C and H_2_:CO of 1:1, and Fe_*x*_NC/AlFe at 270 °C and H_2_:CO of 2:1 yield nearly 60 wt % of their total reactor mass in the C_10_ fraction. This suggests that the susceptibility to carbon growth on the NiFe and AlFe surfaces during the FTS reactions is facilitated in the chain termination with desorption at the ten-carbon limit. In contrast, the Fe_*x*_NC/CoFe is significantly less prone to this anomaly. While a C_10_ spike was present, the Co formulation at 240 °C and H_2_:CO of 2:1 yields only 6.5 wt % C_10_, bypassing the pore constraints observed in the other catalysts.

The availability of hydrogen acts as the chemical terminating agent for the growing alkyl chains, but its effect on the C_10_ anomaly is more dependent on the nature of the active metal. For Fe_*x*_NC/AlFe at 270 °C, increasing the H_2_:CO ratio from 1:1 to 2:1 increases C_10_ production, driving its yield from 40.0 to 58.5 wt %. For Fe_*x*_NC/CoFe at 270 °C, increasing the hydrogen ratio gives minor internal steric constraints, and the elevated hydrogen partial pressure simply terminated the chains earlier in the growth cycle, shifting the mass balance toward other products.

The calculated ASF kinetics, when mathematically corrected to account for the empirically observed C_10_ selectivity, allows the projection of the full-spectrum hydrocarbon distribution. Table 6 presents the theoretical product proportions (wt %) categorized into standard industrial cuts: light gases (C_1_–C_4_), naphtha (C_5_–C_8_), sustainable aviation fuel (SAF), precursors (C_9_–C_14_), diesel (C_15_–C_20_), and heavy waxes (C_21+_). Analyzing these discrete fractions reveals that differences in selectivity of the catalyst are driven by this structure, temperature, and syngas composition.

**Table 6:** Hydrocarbon Product Proportions (wt %) for the Evaluated Catalysts at Varying Temperatures and H_2_:CO Molar Ratios

catalyst	temperature (°C)	H_2_:CO molar ratio	light gases (C_1_–C_4_)	Naphtha (C_5_–C_8_)	SAF (C_9_–C_14_)	diesel (C_15_–C_20_)	heavy waxes (C_21+_)
Fe_*x*_NC/MgFe	240	1:1	45.1	31.7	19.9	2.8	0.5
Fe_*x*_NC/MgFe	270	1:1	1.6	3.3	52.7	6.9	35.6
Fe_*x*_NC/MgFe	240	2:1	10.7	15.3	53.3	10.6	10.1
Fe_*x*_NC/MgFe	270	2:1	33.0	27.5	34.6	3.9	1.0
Fe_*x*_NC/NiFe	270	1:1	9.0	11.7	69.0	6.0	4.3
Fe_*x*_NC/NiFe	240	2:1	96.5	3.1	0.4	0.0	0.0
Fe_*x*_NC/AlFe	240	1:1	2.1	4.4	36.6	9.4	47.6
Fe_*x*_NC/AlFe	270	1:1	22.2	20.8	51.4	4.2	1.4
Fe_*x*_NC/AlFe	240	2:1	15.2	19.1	50.8	9.0	5.9
Fe_*x*_NC/AlFe	270	2:1	4.6	7.5	67.5	8.0	12.4
Fe_*x*_NC/CoFe	270	1:1	33.5	21.8	42.9	1.6	0.3
Fe_*x*_NC/CoFe	240	2:1	69.3	21.1	9.4	0.2	0.0
Fe_*x*_NC/CoFe	270	2:1	48.8	19.2	31.7	0.3	0.0
Fe_*x*_NC/LiFe	270	1:1	94.1	4.4	1.4	0.0	0.0
Fe_*x*_NC/LiFe	270	2:1	97.2	1.3	0.3	0.0	0.0
Fe_*x*_NC/TiFe	270	1:1	67.7	17.7	14.4	0.1	0.0
Fe_*x*_NC/TiFe	240	2:1	96.1	3.4	0.4	0.0	0.0

Consistent with its classical FTS kinetic profile, the Fe_*x*_NC/CoFe shows a preference for lighter fractions, producing negligible amounts of heavy waxes under all conditions. In a hydrogen-rich environment at lower temperatures at 240 °C and H_2_:CO ratio of 2:1, Fe_*x*_NC/CoFe would direct 69 wt % of its total product mass to light fractions. Even under conditions that maximized its chain growth at 270 °C and H_2_:CO ratio 1:1, over 55% of the product spectrum would remain in the light gas and naphtha cuts.

The most notable feature of the product distribution is the high yield of the SAF precursor fraction across compositions. In these highly constrained catalysts, the middle distillate fraction would dominate the mass balance. Fe_*x*_NC/NiFe at 270 °C and H_2_:CO ratio 1:1 and Fe_*x*_NC/AlFe at 270 °C and H_2_:CO ratio of 2:1 would direct 69 and 67 wt % of their total mass, respectively, into this specific aviation-range window. The majority of the mass within this SAF cut is concentrated in a near-single-component product stream.

While the Fe_*x*_NC/MgFe and Fe_*x*_NC/AlFe are susceptible to funneling mass into the targeted SAF fraction under specific conditions, they simultaneously serve as the primary wax producers if the intrinsic propagation kinetics is unhindered. The Fe_*x*_NC/AlFe operating at 240 °C and H_2_:CO ratio of 1:1 represents the upper limit of chain propagation in this study. The Fe_*x*_NC/MgFe at 270 °C and H_2_:CO ratio of 1:1 similarly demonstrates significant heavy-end selectivity, yielding 35 wt % waxes.

The significant variations in chain-growth probability (α) and product selectivity can be attributed to the role of the Mg, Al, Ni, or Co promoters within the Fe_*x*_NC/MFe catalysts. As previously reported for similar systems, adding an Mg promoter to Fe_*x*_NC/MgFe accounts for the strong metal-promoter interaction, which alters the surface H/C ratio during the reaction. By facilitating strong CO adsorption while competitively restricting hydrogen dissociative adsorption, the Fe_*x*_NC/MgFe significantly suppresses chain termination. This mechanism corroborates the data presented in Table 5, where the Fe_*x*_NC/MgFe exhibits high chain-growth probabilities and a strong bias toward wax production. A similar structural hindrance is likely responsible for the elevated α and C_10_ shape-selective retention observed in the Fe_*x*_NC/AlFe counterpart.

The incorporation of promoters such as cobalt and nickel into the Fe_*x*_NC matrix altered the reaction pathway. Rather than acting purely as structural modifiers like Mg or Al, these elements actively participated in the hydrogenation kinetics. The distinctive behavior of the Fe_*x*_NC/CoFe can be elucidated by its inherent FTS activity, yielding more stable and predictable product distributions than the Fe_*x*_NC/NiFe counterpart. Additionally, the structural and electronic stabilization provided by the FeCo alloy in Fe_*x*_NC/CoFe accelerated chain-termination rates, driving the chain-growth probability to a stable range of 0.55–0.68, in agreement with literature observations.^61,62^ Consequently, the product distribution is shifted toward light gases and middle distillates, actively bypassing the pore-constrained C_10_ trap and the heavy-wax regime characteristic of the iron matrix alone. In contrast, the Fe_*x*_NC/NiFe departs from the heavy-wax regime, while its catalytic performance lacks the environmental stability of the Fe_*x*_NC/CoFe system. The incorporation of nickel into the Fe-based catalyst usually promotes strong synergistic effects,^63^ but these effects manifest as a high kinetic volatility. While the Fe–Ni synergy is kinetically powerful, it lacks the environmental stability of the cobalt-modified system. This accelerated reduction elucidates the high sensitivity observed in the Fe_*x*_NC/NiFe product distributions. Under lower hydrogen availability at 270 °C and H_2_:CO ratio of 1:1, this alloy effectively funneled 69 wt % of the mass into the SAF precursor cut. However, because highly reduced Fe–Ni alloys inherently possess strong methanation capabilities, exposing this easily reducible catalyst to a hydrogen-rich environment at 240 °C and H_2_:CO ratio of 2:1 results in a complete kinetic collapse. The elevated hydrogen partial pressure capitalized on the enhanced carburization rate, driving the chain-growth probability to near zero and converting the entire liquid product spectrum to light products.

From a fundamental thermodynamic perspective, increasing the reaction temperature generally enhances desorption rates in the FTS reaction, thereby theoretically shifting the intrinsic product distribution toward lighter fractions.^57,59,65^ However, the product distribution in these promoted matrices is modulated by internal mass transfer rather than by pure surface thermodynamics. While Fe_*x*_NC/NiFe demonstrates high selectivity toward middle distillates under specific conditions, Fe_*x*_NC/AlFe and Fe_*x*_NC/MgFe display the highest propensity for synthesizing heavy hydrocarbons. This shift toward the heavy regime is intrinsically linked to intraparticle phenomena; details on how gas–liquid solubilities, wax dissolution, and internal diffusion control dictate the extent of chain growth during FTS operational conditions have been extensively established in previous works.^66,67^

Differences among the observed catalytic properties could be attributed to the type of promoter forming the MFe_2_O_4_ phase (M=Ni or Co) and the interaction of nitrogen–carbon-supported MFe_2_O_4_ composite. This effect is more prominent at the highest FTS reaction temperatures for Fe_*x*_NC/NiFe and Fe_*x*_C/CoFe, where the synergistic presence of a carbonaceous interface between the surface iron carbide/nitride and the Co in Fe spinel oxide prevents the carburization extents on Fe_*x*_C and FeN_*x*_ phases, in a similar manner as found elsewhere.^68^ Under lower hydrogen availability at 270 °C and H_2_:CO ratio of 1:1, there is a regulating effect stabilizing the active θ-Fe_3_C, Fe_*x*_N, and χ-Fe_5_C_2_, which enables the Fe_*x*_NC/NiFe to efficiently trap the middle distillates and yield 69 wt % SAF precursors. Therefore, it provides a barrier against the metallic Fe or θ-Fe_3_C and χ-Fe_5_C_2_ iron carbide/nitride active phase and a further oxidation of the former, which determines the best performance of the catalysts. Comparable influences of the carbon species covering Fe carbides for the FTS reaction assume a positive role in enhancing the FTS reaction, in line with the findings.^25^ However, as previously established, this protective interface is overcome by the inherent methanation activity of the Fe–Ni alloy when the catalyst is exposed to the highly reducing, hydrogen-rich environment at 240 °C and H_2_:CO ratio of 2:1.

In the case of Fe_*x*_NC/LiFe, a rapid carburization elucidates the low intrinsic chain-growth probability calculated for this catalyst. The highly reducible active sites heavily favor early chain termination, generating a primarily light-fractional intrinsic distribution before encountering the physical pore constraints at the C_10_ boundary.

Besides, the selectivity to the products also reflects the graphitic carbon encapsulating Fe spinel composite promoted by N strengthening the Fe–C or Fe–N bond with the increment in the electron density on Fe species while weakening Fe–H and C–O bonds, as found elsewhere.^56,68–70^ Noteworthy, these facts contribute to the enhanced CH_*x*_ groups on the solid surface and their further transformation into olefins, and with a notable rise in the longer-chain hydrocarbon molecules. Moreover, methane selectivity is very low, regardless of the other light olefins. Even though the findings state that the χ-Fe_5_C_2_ phase produces methane selectively, the θ-Fe_3_C hinders methane formation.^68–70^

The phase transformation for Fe-based phases such as Fe_3_O_4_, γ-Fe_2_O_3_, and α-Fe_2_O_3_, especially in Fe_*x*_NC/NiFe, Fe_*x*_NC/TiFe, and Fe_*x*_NC/MgFe, is the leading cause of catalyst deactivation, as shown by the XRD results and spent catalyst characterizations (Figure 8a).

**Figure 8:**
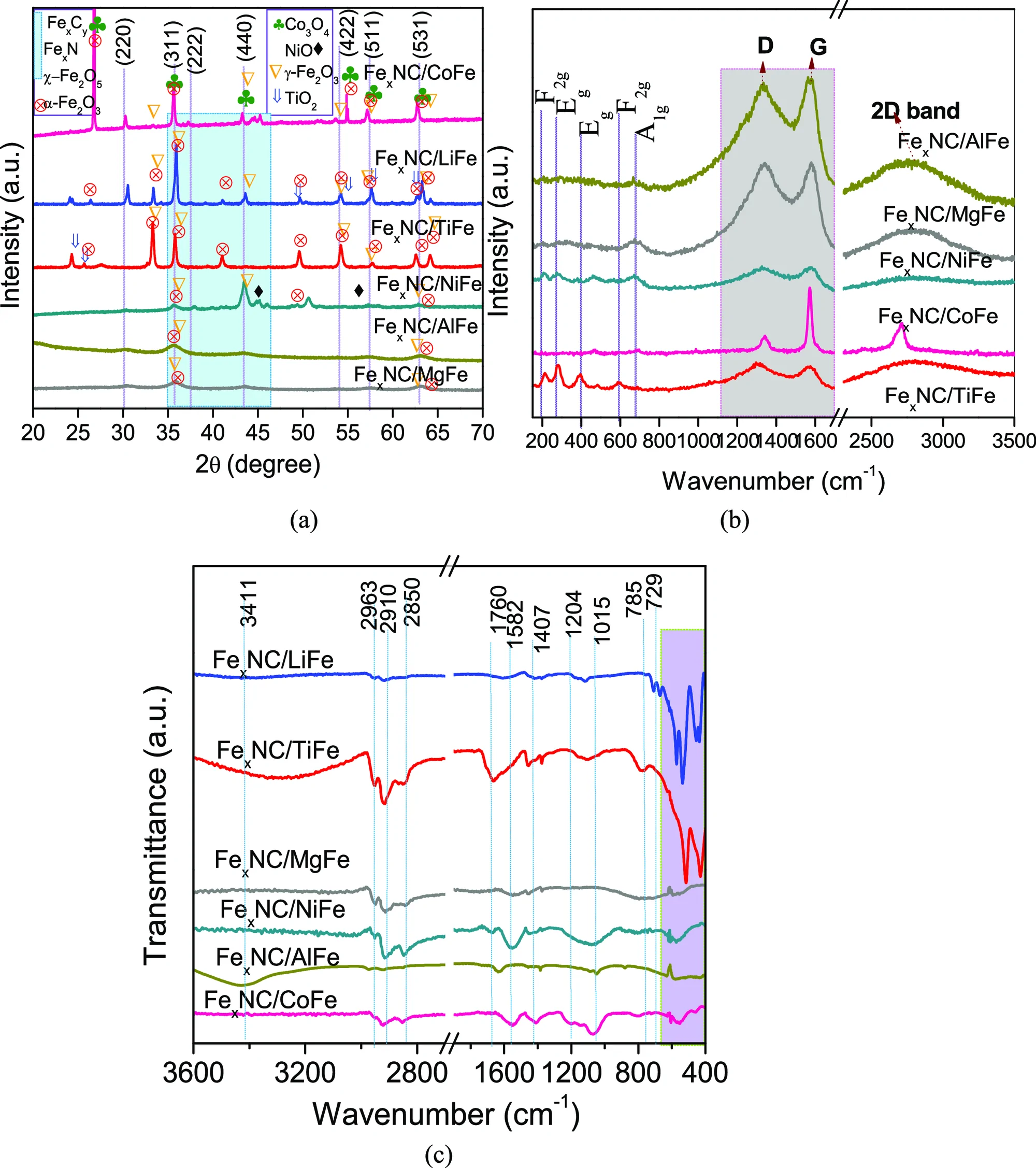
Spent catalyst characterizations of the composites: (a) XRD patterns, (b) Raman and (c) ATR-FTIR spectra.

Raman spectra of the spent solids confirm these assumptions, showing the modes at 198 (F_2g_), 310 (E_g_), 420–487 (E_g_), 543 (F_2g_), and 676 cm^–1^ (A_1g_), while the two-magnon scattering appears at around 1310 cm^–1^ (Figure 8b). All of them were assigned to the Raman modes of magnetite, besides appearing concomitantly with the D and G bands (highlighted in gray) in addition to the 2D bands associated with carbon species of the carburized solids, after the reaction, in agreement with the findings.^15,48^ Thus, deactivation of the Ti-promoted solid is likely due to the metallic Fe reoxidation to Fe_3_O_4_ by H_2_O, concomitantly, the formation of anatase TiO_2_ species (Figure 8a), despite the introduction of titanium. Most importantly, the Li-promoted Fe spinel-like phase on the nitrogen-doped carbon catalyst is slightly more active than Fe_*x*_NC/MgFe for the FTS reaction.

Although the FTS reaction is a structure-sensitive reaction with the Fe particle sizes affecting the catalytic performance,^65,68–70^ a direct correlation with the crystallite sizes influencing the catalytic properties of the solids is not observed. XPS results reveal that the Fe active sites are highly dispersed on the solid surface, accounting for nearly identical accessibility to the reactants for all solids. Furthermore, the nanosized iron ¨carbides and nitrides on solid surfaces are the dominant active phases, with the promoters helping in the Fe spinel-like phase stabilization to avoid phase transformation over the course of the reaction. Moreover, the increment in the temperature from 240 to 270 °C over Fe_*x*_NC/MFe does not enhance their catalytic performances significantly.

ATR-FTIR spectra of the spent solids in Figure 8c show a prominent absorption band at around 3411 cm^–1^ for Fe_*x*_NC/TiFe and Fe_*x*_NC/AlFe, which is associated with the δ­(O–H) deformation vibrations. Other bands at 2963, 2910, and 2852 cm^–1^ are attributed to the ν­(C–H) symmetrical and asymmetrical stretchings in CH_2_ and CH_3_ groups of alkanes.^71^ At around 1760, 1582, and 1407 cm^–1^, the absorption bands of ν­(C–H), C=C bond vibrations in alkenes, and the CH_2_ and CH_3_ aliphatic groups scissoring vibrations appear in the spectra, in agreement with the literature.^72^ The absorption bands at 1204 and 1015 cm^–1^ are attributable to the vibrations of benzene derivatives. Moreover, some of the low-frequency bands, in particular, at 785 and 729 cm^–1^ are assigned to the CH_2_ rocking vibration of cis-disubstituted olefins, while the CH_2_, −CH, −CH_3_, R–CH=CH–R, (CH_2_)*n* > 4 groups vibrations arise below 600 cm^–1^ owing to the presence of heavy hydrocarbons.^71^ The ATR-FTIR spectra confirm the occurrence of the products obtained in Table 6, as a result of the chemisorption of waxes on the solid surfaces, after the reaction. This is in line with the findings that illustrate the occurrence of long-chain paraffins in FTS reactions conducted at low temperatures due to the accumulation of these entities in the pores of the solids.^72^

The nitrogen-doped graphitic carbon layers encapsulating the Fe spinel phase promoted with Ni result in a synergy among these entities owing to their strong surface interactions with the support, resulting in a defective structure in a stable catalyst to produce light olefins and alcohol during the FTS reaction. A comparison of the catalytic performance of various Fe-containing carbides and Fe-nitride-doped carbon catalysts reported for the syngas conversion via the FTS reaction is given in Table 7.

**Table 7:** Comparison of Various Fe-Based Catalysts for FTS Reaction at Distinct Conditions of Temperatures and CO to H_2_ Molar Ratios^a^

catalyst	reaction conditions	X(%)	refs
FeA/CSs-B-IM	*t* = not described. *P* = 8 atm. *T* = 275 °C; H_2_/CO = 2.0, GHSV = 2760 h^–1^ in a fixed bed-type reactor. Selectivity: CH_4_ = 20%, C_2_–C_4_ olefins = 22%, C_5_^+^ = 57%	0.16^b^	^55^
Fe_5_C_2_/NC600	*t* = 0.5 h. *P* = 1.8 atm. *T* = 250 °C; H_2_/CO = 2.0, GHSV = 54 000 h^–1^ during a photothermal FTS reaction in a batch-type reactor. Selectivity: CH_4_ = 22%, C_2_–C_4_ olefins = 55%, C_2_–C_4_ paraffins = 6% C_5_^+^ = 16%	22	^57^ (and references herewith)
K–Fe/NC	*t* = 24 h. *P* = 29.6 atm. *T* = 240 °C; H_2_/CO = 2.0, GHSV = 4000 h^–1^ in a fixed bed reactor. Selectivity: alcohol = 30%; C_2_–C_13_ fraction = 95%.	21	^69^
CNF-supported Fe	*t* = 20 h. *P* = 20 atm. *T* = 340 °C; H_2_/CO = 1.0, GHSV = 54 000 h^–1^ in a fixed bed reactor. Selectivity: CH_4_ = 8%, C_2_–C_4_ olefins = 60%, C_2_–C_4_ paraffins = 5% C_5_^+^ = 27%	16	^70^
Fe_*x*_NC/NiFe	*t* = 6 h. *P* = 20 atm. *T* = 270 °C; H_2_/CO = 1.0, GHSV = 13.5l NTP. g_cat_^–1^ h^–1^ in a batch-type reactor. Selectivity: CH_4_ = 2%, C_2_–C_4_ olefins and C_2_–C_4_ paraffins = 5%; C_6_–C_9_^+^ fraction = 93%.	44	this work

a X denotes the CO conversion; *t* is the reaction time; *T* is the reaction temperature; *P* is the pressure of the system; and GHSV is the gas hourly space velocity.

b The CO conversion for FeA/CSs-B-IM is not informed, and the value is for the metal time yield in mol_CO_ g_Fe_^–1^ h^–1^. C_2_–C_4_ olefins are olefins with carbon numbers of 2–4; C_2_–C_4_ paraffins are the paraffins with carbon numbers of 2–4. C_5_^+^ refers to the hydrocarbons with carbon numbers above 5; C_2_–C_13_ are paraffins with carbon numbers of 5–11; and C_10_^+^ are the hydrocarbon products with more than ten carbons.

Most literature reports require relatively elevated pressures, high temperatures, and long-term reaction runs to achieve moderate to high CO conversions in FTS. It is evident that the catalytic performance offered by Fe_*x*_NC/NiFe is good CO conversion of ca. 44% under much milder conditions, e.g., 270 °C, 20 atm, and a shorter reaction time of 6 h, exhibiting its comparable catalytic activity with regard to previously reported systems.

It is found that χ-Fe_2_O_5_, θ-Fe_3_C, and Fe_3_N carbides (highlighted in the light blue square in Figure 8a), in addition to Fe_3_O_4_, γ-Fe_2_O_3_, and α-Fe_2_O_3_, after the FTS reaction, could act as active carbide phases (Figure 8a). Raman spectra of spent Fe_*x*_NC/NiFe corroborate these results with the Raman bands at around 1320 (D band) and 1550 cm^–1^ (G band). These bands are assigned to the disordered graphitic structures and *sp* ^3^-bonded carbons, while the band near 1550 cm^–1^ (Figure 8b) can be due to the *sp* ^2^-bonded carbons, as found elsewhere.^48^ The intensity of the G band decreases in all samples, indicating the carburization of the solids after the catalytic runs. Moreover, the carbonaceous matrix with Ni as promoter is stable, favoring the kinetics and lowering the barrier to the formation of the active carbide phase, decreasing the number of heavy hydrocarbons.

## 4 Conclusion

The nitrogen-doped carbon encapsulation of Fe_3_O_4_, γ-Fe_2_O_3_, or other spinel Fe phases resulted in the formation of multifunctional porous structures. The addition of heteroatoms in the nitrogen-doped carbon-supported Fe_3_O_4_ composites influenced the catalytic performance in the syngas conversion via Fischer–Tropsch reaction. The Ti and Al addition into nitrogen-doped carbon Fe composite had a negligible catalytic performance owing to their deactivation by phase transformations. The existence of surface iron carbides and iron nitrides covering a carbon matrix strongly bonded to the Fe^2+^/Fe^3+^ entities from the Fe spinel phase, besides introducing defects into the structure, was observed when Mg or Li is added as a promoter. The improved selectivity for olefins and paraffins, in contrast to the production of light and heavy hydrocarbons, is observed for Ni, Fe, and carbides and nitrides synergistic effects in the nitrogen-doped carbon-supported NiFe catalyst during FTS at distinct reaction conditions.
